# Dose-dependent effects of electron beam irradiation on dental materials: a systematic review of *in vitro* evidence

**DOI:** 10.3389/fdmed.2026.1871959

**Published:** 2026-08-11

**Authors:** Shreya Chandrashekar, Niranjan Harikrishna, Avishikta Banerjee, Chaithra Lakshmi V, Nishmitha N. Hegde, Mithra N. Hegde

**Affiliations:** Department of Conservative Dentistry and Endodontics, AB Shetty Memorial Institute of Dental Sciences, NITTE (Deemed to be University), Mangaluru, India

**Keywords:** composite resins, dental materials, dose-response, electron beam irradiation, *in vitro*, mechanical properties, surface characteristics, systematic review

## Abstract

**Background:**

Electron beam irradiation (EBI) is a non-chemical technique proposed for modifying dental materials, but evidence is contradictory. Some studies report gains in mechanical performance, others document discoloration, structural breakdown, or cytotoxicity at higher doses.

**Objectives:**

To systematically review effects of EBI on mechanical strength, surface characteristics, chemical stability, and biological responses in dental materials, and identify dose ranges where effects shift from beneficial to harmful.

**Methods:**

PubMed, Scopus, and Web of Science were searched for laboratory studies published January 2001–March 2026 using the terms “electron beam irradiation” and “dental materials.” Two reviewers independently screened records; a third resolved disagreements. Eligible studies were laboratory-based experiments evaluating dental materials under EBI. Data were extracted on post-irradiation mechanical, physicochemical, biological, and appearance outcomes. Risk of bias was assessed using RoBDEMAT and QUIN; certainty was rated using a modified GRADE approach.

**Results:**

Of 897 records identified, 18 studies met inclusion criteria. Materials tested included composites, nanocomposites, fiber-reinforced composites, denture base polymers, orthodontic bracket materials and adhesives, alloy-resin bonding systems, monolithic zirconia, acrylic resins, glass ionomer cement, dental irrigants, cast titanium, and zinc oxide eugenol. Doses ranged from 1 to 200 kGy, with acceleration energies of 25 kV–10 MeV (two studies did not report beam energy). At low-to-moderate doses, most polymer-based materials showed improved flexural strength, fracture toughness, Vickers hardness, and wear resistance, whereas higher doses caused polymer degradation, discoloration, loss of structural integrity, and genotoxic/cytotoxic effects. QUIN classified 4 studies (22%) as low risk, 12 (67%) as medium risk, and 2 (11%) as high risk of bias.

**Limitations:**

All evidence was *in vitro*; studies were heterogeneous in materials, doses, and outcomes; irradiation parameters were inconsistently reported; modified GRADE certainty was very low across all outcomes.

**Conclusions:**

Current *in vitro* evidence indicates EBI modifies dental material properties, with mechanical improvements most consistent for dimethacrylate-based polymer systems at low-to-moderate doses (1–100 kGy), while color instability and dose-dependent degradation remain important barriers. Because the evidence base is entirely *in vitro* and of very low certainty, findings are preliminary; dose-optimization and *in vivo* studies are needed before clinical recommendations can be made.

**Systematic Review Registration:**

https://doi.org/10.17605/OSF.IO/HAVT8.

## 1 Introduction

Ionizing radiation techniques such as electron beam and gamma irradiation have become effective tools for modifying the physical, mechanical, and chemical characteristics of polymers and composites used in dentistry (1). These techniques have also gained increasing attention as a promising modification method capable of altering the structural and surface characteristics of polymers and ceramics without requiring chemical additives or catalysts (2). The use of advanced dental restorative and biomedical materials such as resin-based composites, glass ionomer cements, denture base polymers, endodontic sealers, and surface-modified titanium has become central to modern dentistry for functional and aesthetic rehabilitation.

Electron beam irradiation is a high-energy ionizing radiation technique that generates accelerated electrons capable of penetrating and interacting with matter at the molecular level (3). Depending on the dose and energy settings, EBI can induce cross-linking, chain scission, oxidation, and surface functionalization in polymeric systems; these mechanisms are relevant to the mechanical, chemical, and biological behavior of dental substrates. These transformations have been exploited in various biomedical domains, including sterilization, surface activation, and scaffold engineering (4).

The first application of EB irradiation to dental materials was reported by Behr et al. in 2005, who investigated its effects on dental composites (5) and alloy-to-resin bond strength (6). Since then, multiple research groups have examined the effects of EB irradiation on a diverse range of dental materials, including resin composites, nanocomposites (5, 7–9), fiber-reinforced composites (10, 11), denture base polymers (12), orthodontic bracket materials (13, 14), acrylic resins (15), monolithic zirconia (16), cast titanium (17), glass ionomer cements (18), dental irrigants (19), root canal sealers (20), and monomer leaching behavior (21).

Despite nearly two decades of research, the available evidence has not been systematically synthesized. Individual studies have used varying irradiation parameters, tested different material types, and assessed diverse outcome measures, making it difficult to draw overarching conclusions. Furthermore, no previous systematic review has applied validated risk of bias tools specifically designed for dental materials research, nor has the certainty of evidence been formally evaluated for this body of literature. This systematic review aimed to systematically identify, critically appraise, and qualitatively synthesize all available *in vitro* evidence on the effects of electron beam irradiation on dental materials, encompassing mechanical, physical, chemical, and biological properties, using validated assessment tools and transparent methodology. This review follows PRISMA 2020 reporting guidelines (22).

## 2 Methods

### 2.1 Protocol and registration

This review was not registered prospectively, and no a-priori protocol was filed on a public registry before the review began; the protocol was developed in parallel with the conduct of the review and registered retrospectively on the Open Science Framework (OSF; https://doi.org/10.17605/OSF.IO/HAVT8) after the database search and screening were completed. The review is reported following the PRISMA 2020 statement (22), and the completed PRISMA 2020 checklist is provided as Supplementary Material S1. Two deviations from the registered OSF record are noted and justified: (i) an erroneous authorship-overlap entry for Shetty 2014 was corrected, as that study does not in fact share authorship with the review team; and (ii) one further eligible study (Ravi et al., 2014) identified during peer review was added. The OSF record has been updated to match, and the retrospective nature of the registration is acknowledged as a limitation in Section 4.8.

### 2.2 Eligibility criteria

**Inclusion criteria** were as follows: (1) *In vitro* laboratory studies evaluating the effect of electron beam irradiation on any dental material or dental medicament; (2) studies reporting at least one quantifiable or qualitative outcome measure (mechanical, physical, chemical, optical, or biological property); (3) studies including a non-irradiated control or comparison group; (4) peer-reviewed publications in the English language (applied because validated translation and reliable duplicate data extraction from non-English reports were not feasible within the review team's resources); (5) studies published from January 2001 to March 2026.

#### 2.2.1 Exclusion criteria

(1) Studies using gamma irradiation, UV irradiation, x-ray irradiation, or other forms of ionizing radiation without a dedicated EB irradiation experimental group; (2) studies on non-dental polymers or general industrial materials without a stated dental application; (3) *in vivo* or clinical studies; (4) review articles, conference abstracts, editorials, letters, case reports, and book chapters; (5) studies with insufficient data for extraction.

#### 2.2.2 Rationale for the design and date restrictions

Only *in vitro* experimental studies were eligible because the review question concerns changes in the physicochemical, mechanical, and biological properties of dental materials measured under controlled laboratory conditions, where the irradiation exposure and the material outcome can be related directly and quantitatively. Studies using animal (*in vivo*) models were excluded because they assessed biological responses in living tissue rather than post-irradiation changes in the properties of the dental material itself; three such animal studies were excluded on this basis (Supplementary Table S3). This boundary reflects the scope of the review question and is not a claim that *in vivo* evidence does not exist. Publications were restricted to January 2001–March 2026 to capture the modern body of work on electron-beam processing of contemporary dental materials, with the earliest eligible study published in 2005.

The eligibility criteria were framed using a modified PICOS framework adapted for *in vitro* studies: P (Population): dental materials, dental medicaments, and dental substrates; I (Intervention): electron beam irradiation at any dose and energy; C (Comparison): non-irradiated control specimens; O (Outcomes): mechanical, physical, chemical, optical, or biological properties; S (Study design): *in vitro* experimental studies.

#### 2.2.3 Justification for inclusion of dental irrigants

Two studies (19, 20) evaluated EB irradiation effects on dental irrigants (NaOCl, CHX) and root canal sealers (ZOE), which are pharmacological or cementitious agents rather than structural biomaterials. These were included because: (a) they are classified as dental medicaments within the broader category of dental materials in dental materials science curricula and textbooks; (b) EB irradiation was applied as the independent variable with the explicit aim of modifying their properties for dental applications; and (c) their inclusion provides a comprehensive overview of all dental applications of EB irradiation explored to date. However, it is acknowledged that the physicochemical mechanisms governing EB effects on aqueous solutions and cements differ from those affecting polymeric substrates, and their results are discussed separately in the synthesis.

### 2.3 Information sources and search strategy

A systematic search was conducted across three electronic databases: PubMed/MEDLINE (*N* = 145), Scopus (*N* = 419), and Web of Science (*N* = 333), yielding a total of 897 records. All database searches were conducted on 24 March 2026. The search was limited to English-language publications because the review team did not have the resources to obtain validated translations or to perform reliable duplicate data extraction from non-English reports; this pragmatic restriction is acknowledged as a review-level limitation (Section 4.8). Additionally, the reference lists of all included studies and relevant review articles were manually screened to identify any further eligible studies not captured by the electronic search.

Five studies identified through hand-searching of reference lists (Studies 4, 12, 13, 14, and 17) (7, 8, 18, 20, 21). Table 1 were published in journals with limited or discontinued indexing in PubMed, Scopus, and Web of Science and were therefore not retrieved by the the electronic database searches; these studies overlap in authorship with the review team (see conflict of Interest) and were retained because they met all pre-defined inculsion criteria. Their methodological quality was assessed independently by two non-conflicted reviewers (SC and NH) using the QUIN tool and RoBDEMAT, with risk of bias transparently reported in Tables 2, 3.

**Table 1:** Standardised data extraction chart of included studies (*n* = 18).

Sl.	Author, Year (Ref.)	Design	Material	*n*	Dimensions	Dose (kGy)	Energy (MeV)	Equipment	Comparator	Properties	Testing	Results	Summary	RoB	Funding
1	Shetty AV et al., 2014 (19)	*In vitro*	NaOCl (1.25%, 2.5%), CHX (1%, 2%)	NS	10 mL pouches	1, 2, 5, 10	NS	Microtron, Mangalore Univ.	Non-irradiated NaOCl/CHX (0 kGy)	Antimicrobial, pH, cytotoxicity, stability	Disc diffusion, MTT, ANOVA	1.25%NaOCl + 1 kGy vs. E.faecalis: 16 ± 0.0 mm (ctrl 13.75 ± 0.95); 1%CHX + 1 kGy vs. S.aureus: 26 ± 0.0 mm (ctrl 19 ± 0.0)	EB (1 kGy) ↑antimicrobial activity without altering biocompatibility	Med.	Not reported
2	Behr M et al., 2006 (11)	*In vitro*	Vectris/Targis & Stick/Sinfony FRC	*n* = 8/grp	25 × 4 × 4 mm	100 (3 × 33.3)	10	Rhodotron, BGS, Germany	Non-irradiated specimens; thermocycled (TCML) group	Fracture resistance	Restrained-end 3 pt bend, Zwick, ANOVA, Tukey	Stick/Sinfony: Irr 520 ± 31 N vs. TCML 362 ± 41 N; Vectris/Targis: Irr 575 ± 57 N vs. TCML 383 ± 51 N	EB ↑fracture load; prevented facing fractures during TCML	Low	Not reported
3	Faltermeier A et al., 2007 (12)	*In vitro*	6 experimental denture base polymers (MMA, MMA/EGDMA, MMA/BDDMA)	*n* = 10/sub	36 × 8 × 4 mm	25, 200	4.5	Rhodotron, BGS, Germany	Non-irradiated (0 kGy) blends	K1c, FW, VH, colour	3 pt bend, VH, spectrophotometer, ANOVA	25 kGy: K1c↑ significant; 200 kGy: all↓; *Δ*E > 3 all blends	Low-dose improved; unacceptable colour changes	Low	Not reported
4	Hegde MN et al., 2014 (7)	*In vitro*	Filtek Z350 XT & Ceram X-duo	*n* = 12/grp	25 × 2 × 2 mm ISO4049	1, 3, 5	8	Microtron, Mangalore Univ.	Non-irradiated control (0 kGy)	Flexural strength	3 pt bend, Instron, ANOVA, Tukey	Z350XT: Ctrl 170.89 ± 9.07; 5 kGy 377.64 ± 74.5 MPa (*p* < 0.001); CeramX: Ctrl 120.14 ± 7.12; 5 kGy 229.33 ± 59.15	EB ↑FS dose-dependently	Med.	Not reported
5	Behr M et al., 2005 (6)	*In vitro*	Ti gr.I, CoCr with Rocatec, MetalPrimerII, SR Link	*n* = 10/sub	20 × 10 × 2 mm plates	100 (5 × 20)	10	Rhodotron, BGS, Germany	Non-irradiated control; thermocycled group	SBS, TS, fracture mode	ISO TR 11405, Zwick, ANOVA, Tukey, t-test	MetPrimII Ti SBS: irr 31.9 ± 6.1 vs. ctrl 18.3 ± 3.1 (*p* < 0.001); TS CoCr: irr 40.8 ± 2.2 vs. ctrl 18.9 ± 1.2	Thiol-phosphate + EB: 3–4× ↑bond; stable after TC	Low	Not reported
6	Faltermeier J et al., 2012 (14)	*In vitro*	Brackets: Aesthetik-Line(PC), Brillant(POM), Envision(PU), Exper1&2(UDMA/SiO2)	*n* = 10/grp	Bracket specimens	100	10	Rhodotron, BGS, Germany	Non-irradiated brackets (0 kGy)	VH, colour (*Δ*E)	VH Zwick, spectrophotometer, Wilcoxon	Exper2 VH: irr 22.4 ± 1.03 vs. ctrl 20.80 ± 1.17 (*p* = 0.005); Brillant↓; *Δ*E: Exper2 2.95	VH↑ most except POM; colour changes; 40%SiO2 acceptable	Med.	Not reported
7	Behr M et al., 2005 (5)	*In vitro*	5 composites: ArtGlass, BelleGlass, Sinfony, Solidex, Targis	*n* = 10/sub	36 × 8 × 4 mm; 1 mm(wear)	25, 100, 200	10	Rhodotron, BGS, Germany	Non-irradiated control (0 kGy)	K1c, FW, VH, wear, colour	3 pt bend, VH, ACTA wear, spectrophotometer, Mann–Whitney	*Δ*E > 3 all doses; K1c↑ (*p* < 0.05); wear↓ 100 kGy	EB↑ all mech. properties; simple-cure needed higher doses	Low	Not reported
8	Kolbeck C et al., 2007 (10)	*In vitro*	Construct(PE), everStick(glass/BisGMA/PMMA), FibreKor(glass/BisGMA/PCDMA)	*n* = 8(fr); *n* = 4(SBS)	25 × 2 × 2 mm; 20 × 10 × 2 mm	15, 33, 100(fr); 15, 33(SBS)	10	Rhodotron, BGS, Germany	Non-irradiated control (0 kGy)	Fracture strength, elongation, SBS	Restrained-end UTM, ISO 4049, ISO 11405, Mann–Whitney	FibreKor: 0 kGy 74 N → 100 kGy 231 N (*p* = 0.001); SBS FibreKor + SR-Link 15 kGy (*p* = 0.038)	Fracture↑ all FRCs; SBS↑ specific combos	Low	Not reported
9	Faltermeier A et al., 2011 (13)	*In vitro*	POM, PC, PU bracket materials	*n* = 30/grp	36 × 8 × 4 mm	100	10	Rhodotron, BGS, Germany	Non-irradiated control (0 kGy)	K1c, VH, 3-medium wear	3 pt bend, VH, ACTA wear, Mann–Whitney, ANOVA	PC, PU: K1c↑ (*p* = 0.005), VH↑; POM: K1c↓ (*p* = 0.047); PU wear↓ (*p* = 0.007)	PC & PU improved; POM deteriorated	Low	Not reported
10	Alshamrani & De Souza, 2020 (16)	*In vitro*	5Y-PSZ HT, 3Y-PSZ LT zirconia	*n* = 10(FS), 15(fat), 5(TP)	14 × 4.0 × 1.5 mm ISO6872	0.07 (70 Gy)	6	Elekta linac, Toronto	Non-irradiated zirconia (0 Gy)	FS, fatigue, TP, XRD, SEM	3 pt bend Instron, staircase fatigue, spectrophotometer, ANOVA, Tukey	LT: FS ctrl 712.45 ± 77.04 vs. irr 588.40 ± 143.82 (*p* < 0.05); HT NS; Fatigue & TP NS; t→c ∼20 wt%	Radiation affected LT FS and crystalline content	Low	Not reported
11	Ito K et al., 2014 (15)	*In vitro*	Self-curing acrylic (Unifast III)—powder irradiated	*n* = 10/grp	50 × 5 × 2 mm	25, 50, 75, 100	0.110 (110 kV)	LEB, Hamamatsu Photonics	Non-irradiated control (0 kGy)	FS, E-mod, ESR, mol. weight	3 pt bend Shimadzu, ESR, HPLC, ANOVA, Tukey-Kramer	FS: Ctrl 61.5 ± 3.0; 50 kGy 73.0 ± 1.9 MPa (*p* < 0.01); Mol.wt: 960 K → 390 K	LEB of powder ↑FS; optimal 50 kGy; cross-linking	Low	Not reported
12	Hegde MN et al., 2016 (8)	*In vitro*	Filtek Z350 XT & Filtek Bulk Fill	*n* = 10/grp	25 × 2 × 2 mm ISO4049	2, 4, 10	8	Microtron, Mangalore Univ.	Non-irradiated control (0 kGy)	Flexural strength	3 pt bend, Instron, ANOVA, Student t	Z350XT: Ctrl 172.05 ± 2.93; 10 kGy 388.60 ± 11.13 (*p* < 0.001); BulkFill: Ctrl 155.26 ± 2.54; 10 kGy 311.85 ± 4.96	FS↑ dose-dependently; Z350XT > BulkFill	Med.	Not reported
13	Hegde MN et al., 2014 (18)	*In vitro*	GIC Type IX (3M ESPE)	*n* = 10/grp	25 × 2 × 2 mm ISO4049	10	8	Microtron, Mangalore Univ.	Non-irradiated control (0 kGy)	Genotoxicity: comet, DNA diffusion	Comet assay, DNA diffusion, Student t	Tail length: Irr 50.82 ± 20.4 vs. NR 24.70 ± 24.70 px (*p* < 0.0001); OTM: 26.51 ± 9.92 vs. 15.39 ± 4.67 (*p* = 0.0203)	EB of GIC ↑DNA damage; chain breakage releasing acids	Med.	Not reported
14	Sajnani A et al., 2014 (21)	*In vitro*	Filtek Z350 XT Restorative & Flowable	*n* = 6/sub	2 × 2 × 2 mm	1, 3, 5	8	Microtron, Mangalore Univ.	Non-irradiated control (0 kGy)	Monomer leaching (BisGMA, TEGDMA)	HPLC, Kruskal–Wallis, Wilcoxon, Mann–Whitney	Min leaching at 3 kGy; BisGMA > TEGDMA; Non-irr max leaching	EB ↓monomer leaching; 3 kGy optimal	Med.	Not reported
15	Tokunaga J et al., 2009 (17)	*In vitro*	Pure Ti JIS type 3	*n* = 5(gloss); *n* = 4(corr)	10 × 15 × 1.4 mm	5.0 J/cm^2^; 20–80 pulses	25 kV	CRS 100, Nagata Seiki, Japan	As-cast Ti, 0 pulses	Glossiness, Ra, corrosion, SEM, XRD	Glossmeter, Surfcorder, anodic polarisation, ANOVA, Tukey	Glossiness: 4.1 → 154 Gs (20 pulses); Ra: 2.34→0.31 µm; corrosion↑	EB effective for Ti polishing; surface melting	Low	Not reported
16	Vaishnavi C et al., 2010 (9)	*In vitro*	Adoro indirect composite (Ivoclar)	*n* = 10/grp	14 × 2 × 2 mm; 6 mmØ2 mm	1	6	Siemens accelerator	Conventional inlay-system curing; non-irradiated control	K1c, wear resistance	3 pt bend Lloyd, wear device, Kruskal–Wallis, Mann–Whitney	K1c: EB 0.102 ± 0.002 vs. Inlay 0.112 ± 0.002 vs. Ctrl 0.064 ± 0.002 (*p* < 0.05)	EB comparable to inlay system	Low	Not reported
17	Hegde MN et al., 2013 (20)	*In vitro* (obs.)	ZOE root canal sealer	NS	Powder/liquid microvials	10	8	Microtron, Mangalore Univ.	Non-irradiated ZOE (0 kGy)	Handling, consistency	Visual/tactile assessment	Irradiated ZOE: thicker, pasty consistency	EB improved handling; qualitative only	High	Not reported
18	Ravi MS et al., 2014 (29)	*In vitro*	3 orthodontic bonding adhesives: Unite (3M self-cure; BisGMA/UDMA/TEGDMA), MIP (3M light-cure), GC Fuji (light-cure GIC)	*n* = 2 (haemolysis); 50 cells/replicate (comet)	8 × 2 × 2 mm	2	NS	Microtron, Mangalore Univ.	Non-irradiated adhesives (0 kGy)	Cytotoxicity (haemolysis); genotoxicity (comet: tail length, %DNA in tail, OTM); apoptosis (DNA diffusion)	Comet assay, haemolysis assay, DNA diffusion, ANOVA, Tukey	%DNA-in-tail ↑ after irr (Unite 8.23→21.66; MIP 7.86→31.17; GC Fuji 8.56→15.85); OTM ↑ all three; haemolysis ↑ MIP & GC Fuji	EB (2 kGy) ↑genotoxicity (DNA damage) and cytotoxicity in orthodontic adhesives	Med.	BRNS, Government of India (grant 2011/34/12/BRNS)

Studies are listed by their Table 1 number; the reference-list citation number is given in parentheses in the Author, Year (Ref.) column. Comparator denotes the non-irradiated reference condition. Funding is reported as stated in each source publication; “Not reported” indicates no funding statement was identified. EB, electron beam; kGy, kilogray; Gy, gray; MeV, megaelectronvolt; K1c, fracture toughness; FW, work of fracture; VH, Vickers hardness; SBS, shear bond strength; TS, tensile strength; FS, flexural strength; TCML, thermal cycling and mechanical loading; TC, thermal cycling; FRC, fibre-reinforced composite; PC, polycarbonate; POM, polyoxymethylene; PU, polyurethane; RoB, risk of bias; NS, not specified; ctrl, control; irr, irradiated; grp, group; sub, subgroup; fr, fracture; obs., observational; Med., medium, *Δ*E, colour difference.

**Table 2:** RoBDEMAT risk of bias assessment of included studies (*n* = 18).

Study	D1: Planning & allocation			D2: Specimen prep		D3: Outcome assessment			D4: Data & reporting	
	1.1	1.2	1.3	2.1	2.2	3.1	3.2	3.3	4.1	4.2
1. Shetty AV et al.	Y	NI	NI	PY	PY	PY	Y	NI	Y	Y
2. Behr M et al.	Y	Y	NI	Y	Y	Y	Y	NI	Y	Y
3. Faltermeier A et al	Y	Y	NI	Y	Y	Y	Y	NI	Y	Y
4. Hegde MN et al	Y	NI	NI	Y	PY	Y	Y	NI	Y	Y
5. Behr M et al	Y	Y	NI	Y	Y	Y	Y	NI	Y	Y
6. Faltermeier J et al.	Y	NI	NI	Y	Y	Y	Y	NI	Y	Y
7. Behr M et al.	Y	Y	NI	Y	Y	Y	Y	NI	Y	Y
8. Kolbeck C et al.	Y	NI	NI	Y	Y	Y	Y	NI	Y	Y
9. Faltermeier A et al.	Y	NI	NI	Y	Y	Y	Y	NI	Y	Y
10. Alshamrani & De Souza	Y	Y	Y	Y	Y	Y	Y	NI	Y	Y
11. Ito K et al.	Y	NI	NI	Y	Y	Y	Y	NI	Y	Y
12. Hegde MN et al.	Y	NI	NI	Y	PY	Y	Y	NI	Y	Y
13. Hegde MN et al.	Y	NI	NI	PY	PY	PY	Y	NI	PY	Y
14. Sajnani A et al.	Y	NI	NI	Y	PY	Y	Y	NI	Y	PY
15. Tokunaga J et al.	Y	NI	NI	Y	Y	Y	Y	NI	Y	Y
16. Vaishnavi C et al.	Y	Y	NI	PY	PY	Y	Y	NI	Y	Y
17. Hegde MN et al.	PN	NI	NI	PN	PN	PN	PN	NI	NI	PN
18. Ravi MS et al.	Y	NI	NI	PY	PY	PY	Y	NI	Y	PY

Items: 1.1 = Control group; 1.2 = Randomisation; 1.3 = Sample size calculation; 2.1 = Standardisation of samples; 2.2 = Identical conditions; 3.1 = Standardised testing; 3.2 = Defined outcomes; 3.3 = Blinding; 4.1 = Statistical analysis; 4.2 = Complete reporting. Judgement: Y = Yes; PY = Probably Yes; PN = Probably No; N = No; NI = No Information.

**Table 3:** QUIN quality assessment of included studies with risk of bias classification (*n* = 18).

Study	C1	C2	C3	C4	C5	C6	C7	C8	C9	C10	C11	C12	Tot	Score%	Risk
Shetty AV et al.	2	0	1	2	2	0	0	2	0	0	2	2	13	54.2%	**Medium**
Behr M et al.	2	0	2	2	2	1	2	2	0	0	2	2	17	70.8%	**Low**
Faltermeier A et al.	2	0	2	2	2	0	2	2	0	0	2	2	16	66.7%	**Medium**
Hegde MN et al.	2	0	1	2	2	1	0	2	0	0	2	2	14	58.3%	**Medium**
Behr M et al.	2	0	2	2	2	1	2	2	0	0	2	2	17	70.8%	**Low**
Faltermeier J et al.	2	0	1	2	2	0	0	2	0	0	2	2	13	54.2%	**Medium**
Behr M et al.	2	0	2	2	2	1	2	2	0	0	2	2	17	70.8%	**Low**
Kolbeck C et al.	2	0	2	2	2	0	0	2	0	0	2	2	14	58.3%	**Medium**
Faltermeier A et al.	2	0	2	2	2	0	0	2	0	0	2	2	14	58.3%	**Medium**
Alshamrani & De Souza	2	2	2	2	2	1	2	2	1	0	2	2	20	83.3%	**Low**
Ito K et al.	2	0	2	2	2	1	0	2	0	0	2	2	15	62.5%	**Medium**
Hegde MN et al.	2	0	1	2	2	0	0	2	0	0	2	2	13	54.2%	**Medium**
Hegde MN et al.	2	0	1	2	1	0	0	1	0	0	1	2	10	41.7%	**High**
Sajnani A et al.	2	0	1	2	2	0	0	2	0	0	2	1	12	50.0%	**Medium**
Tokunaga J et al.	2	0	2	2	2	0	0	2	0	0	2	2	14	58.3%	**Medium**
Vaishnavi C et al.	2	0	2	2	2	0	2	2	0	0	2	2	16	66.7%	**Medium**
Hegde MN et al.	2	0	0	1	1	0	0	0	0	0	0	1	5	20.8%	**High**
Ravi MS et al.	2	0	1	2	2	0	0	2	0	0	2	1	12	50.0%	**Medium**

Scoring: 2 = Adequately specified; 1 = Inadequately specified; 0 = Not specified. Maximum = 24. Risk classification: >70% = Low (green); 50%–70% = Medium (yellow); <50% = High (red). Criteria: C1 Aims; C2 Sample size calculation; C3 Sampling technique; C4 Comparison group; C5 Methodology; C6 Operator details; C7 Randomisation; C8 Measurement method; C9 Assessor details; C10 Blinding; C11 Statistical analysis; C12 Results presentation.

The search strategy combined Boolean operators across three databases. The EB irradiation terms included: “electron beam irradiation” OR “electron beam radiation” OR “e-beam irradiation” OR “e-beam radiation” OR “electron irradiation” OR “electron beam processing” OR “electron beam treatment” OR “electron beam curing” OR “electron beam sterilization” OR “accelerated electron” OR “high energy electron” OR “beta irradiation” OR “ionizing radiation”. These were combined (AND) with dental material terms: “dental material” OR “dental materials” OR “resin composite” OR “composite resin” OR “dental composite” OR “resin-based composite” OR “glass ionomer” OR “glass ionomer cement” OR “PMMA” OR “polymethyl methacrylate” OR “denture base” OR “acrylic resin” OR “dental acrylic” OR “orthodontic bracket” OR “ceramic bracket” OR “fiber reinforced composite” OR “endodontic sealer” OR “root canal sealer” OR “dental cement” OR “titanium implant” OR “dental implant” OR “zirconia” OR “dental zirconia” OR “dental porcelain” OR “dental ceramic” OR “nanocomposite” OR “dental nanocomposite” OR “sodium hypochlorite” OR “chlorhexidine” OR “irrigant”. A third filter for dose-related terms was also applied: “kGy” OR “kilogray” OR “MeV” OR “absorbed dose” OR “radiation dose” OR “dose rate” OR “dose-dependent” OR “irradiation dose”. Database-specific adaptations (e.g., MeSH terms for PubMed, TITLE-ABS-KEY fields for Scopus, TS fields for Web of Science) were applied as appropriate. The complete search strings for each database are provided in Supplementary Table S2.

### 2.4 Study selection

**Figure 1:**
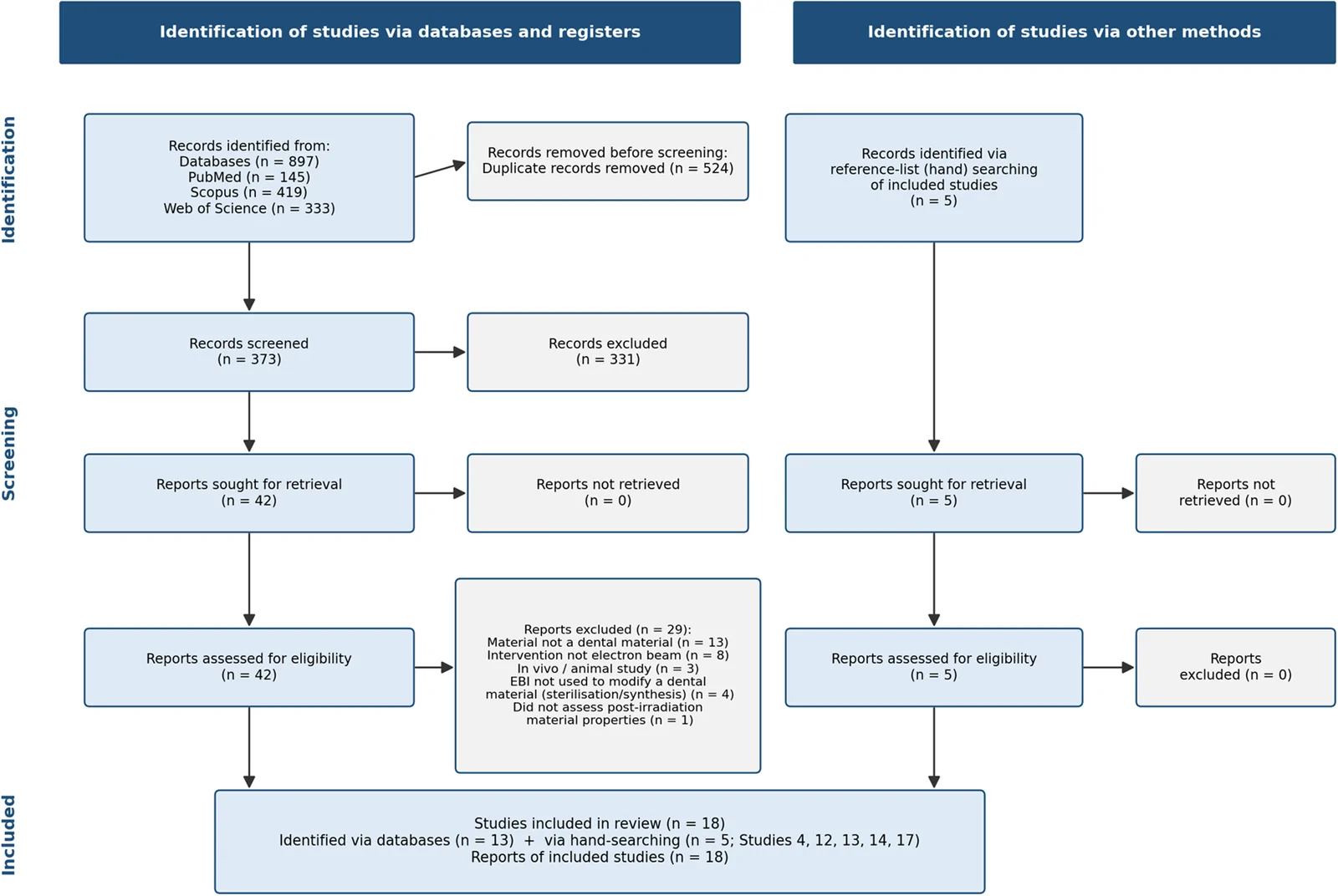
PRISMA 2020 flow diagram.

Two reviewers (SC and NH) independently screened the titles and abstracts of all identified records against the eligibility criteria using Rayyan (rayyan.ai). The same two reviewers then independently assessed the full texts of potentially eligible records. Disagreements at any stage were resolved by discussion and, where necessary, by a third reviewer (NNH). The selection process is summarised in the PRISMA 2020 flow diagram (Figure 1).

The database searches yielded 897 records. After removal of 524 duplicates, 373 records were screened by title and abstract, of which 331 were excluded. The remaining 42 reports were assessed in full text, and 29 were excluded, with the reason for each recorded in Supplementary Table S3: the material studied was not a dental material (*n* = 13); the intervention was not electron beam irradiation (*n* = 8); the study was conducted *in vivo* or in animals (*n* = 3); electron beam irradiation was not used to modify a dental material, for example for sterilisation or synthesis (*n* = 4); and the study did not assess post-irradiation properties of the dental material (*n* = 1). Thirteen studies were included from the database search. A further five studies were identified through reference-list (hand) searching [Studies 4, 12, 13, 14, and 17; references (7, 8, 18, 20, 21)]; all five met the eligibility criteria and were included. In total, eighteen studies were included in the review.

### 2.5 Data extraction

Data were extracted independently by two reviewers (SC and NH), with discrepancies resolved by discussion and, where needed, by a third reviewer (NNH). Risk-of-bias assessment was performed independently by SC and NH using the same arbitration arrangement, and the risk-of-bias section now states these initials explicitly.

The following variables were extracted: author(s) and year of publication, study design, dental material(s) tested (brand name and composition), sample size per group, specimen dimensions and preparation method (including ISO standards followed), EB irradiation parameters (dose in kGy, acceleration energy in MeV, dose rate where reported, number of passes, atmosphere, equipment used), properties evaluated, testing methods and standards, key numerical results (mean ± SD, *p*-values), and summary of main findings. Discrepancies were resolved by consensus. The complete data extraction chart is presented in Table 1.

### 2.6 Risk of bias assessment

Risk of bias was assessed using two tools developed specifically for *in vitro* dental research (formal psychometric validation of RoBDEMAT is limited; it was developed through international Delphi consensus)

(1) **RoBDEMAT** [Dalgado et al. (23)]: A domain-based risk of bias checklist, developed through a Delphi consensus process by 26 international expert stakeholders, with the support of the European Federation of Conservative Dentistry (EFCD) and the Dental Materials Group of the International Association for Dental Research (IADR-DMG). The tool comprises 9 items across 4 domains: D1 (planning and allocation), D2 (specimen preparation), D3 (outcome assessment), and D4 (data treatment and outcome reporting). Each item was judged as Y (Yes/adequately reported), PY (Probably Yes), PN (Probably No), N (No/not reported), or NI (No Information). As recommended by the tool developers, a summary score was not calculated; results are presented as a domain-level checklist (Table 2).
(2) **QUIN Tool** [Sheth et al. (24)]: The Quality Assessment Tool for *in vitro* Studies, developed through a Delphi process with 10 expert panellists comprising 12 criteria, scored as 2 (adequately specified), 1 (inadequately specified), or 0 (not specified). The final quality score was calculated as: QUIN Score (%) = (Total score obtained/Maximum possible score) × 100. Studies were classified as low risk of bias (>70%), medium risk (50%–70%), or high risk (<50%) (Table 3). This tool has demonstrated good content validity (mean score >6/7 from 60 researchers) and reliability (Cronbach's alpha = 0.862) (24).

Both assessments were performed independently by two reviewers (SC and NH). Inter-rater agreement **was calculated using Cohen's kappa.** Disagreements were resolved by discussion and, where necessary, consultation with a third reviewer (NNH).

### 2.7 Certainty of evidence

The certainty of the overall body of evidence was assessed using a modified GRADE (Grading of Recommendations, Assessment, Development and Evaluations) approach (25). Since all included studies were *in vitro* studies, the starting certainty was set as “Very Low” for all outcomes, consistent with the GRADE Working Group's recommendations regarding initial certainty levels for different study designs in preclinical research (26). From this baseline, evidence was assessed across the five standard GRADE domains (risk of bias, inconsistency, indirectness, imprecision, and publication bias) for each outcome category. Evidence was grouped by outcome type rather than by individual study, as recommended by the GRADE framework (27). Upgrading criteria were not applied, as no *in vitro* evidence meets the thresholds for dose-response gradients or large effect sizes for clinical applicability. The certainty assessment was carried out independently by two reviewers (SC and NH), with disagreements resolved by discussion and, where necessary, a third reviewer (NNH). Table 4 presents the modified GRADE assessment.

**Table 4:** Certainty of evidence across outcome categories, rated using a modified GRADE approach.

Outcome	Studies	Risk of Bias	Inconsistency	Indirectness	Imprecision	Pub. Bias	Certainty	Summary of Effect
Flexural strength	5	Not serious	Not serious	Serious^a^	Not serious	Undetected	**Very Low**	EB (1–200 kGy) consistently ↑FS of polymer-based materials
Fracture toughness/resistance	5	Not serious	Not serious	Serious^a^	Serious^b^	Undetected	**Very Low**	Generally ↑; dose-dependent; high doses↓ some materials
Vickers hardness	5	Not serious	Serious^c^	Serious^a^	Not serious	Undetected	**Very Low**	↑ most materials; ↓ POM; material-dependent
Wear resistance	3	Not serious	Not serious	Serious^a^	Serious^b^	Undetected	**Very Low**	Consistently ↓ wear across composites and polymers
Bond strength	2	Not serious	Not serious	Serious^a^	Serious^b^	Undetected	**Very Low**	Thiol-phosphate + EB: 3–4× ↑; stable after TC
Colour stability (*Δ*E)	4	Not serious	Not serious	Serious^a^	Not serious	Undetected	**Very Low**	*Δ*E > 3.3 most materials; 40%SiO2 acceptable
Antimicrobial activity	1	Serious^d^	N/A	Serious^a^	Serious^b^	Undetected	**Very Low**	1 kGy ↑activity without altering biocompatibility
Monomer leaching	1	Serious^d^	N/A	Serious^a^	Serious^b^	Undetected	**Very Low**	3 kGy ↓leaching from nanocomposites
Genotoxicity	2	Serious^d^	Not serious	Serious^a^	Serious^b^	Undetected	**Very Low**	2–10 kGy ↑DNA damage in GIC and orthodontic adhesives (2 studies)
Cytotoxicity	1	Serious^d^	N/A	Serious^a^	Serious^b^	Undetected	**Very Low**	2 kGy ↑haemolysis in MIP and GC Fuji; Unite unchanged
Zirconia mech./optical	1	Not serious	N/A	Serious^a^	Not serious	Undetected	**Very Low**	70 Gy ↓FS of LT; fatigue & TP unaffected
Ti surface polishing	1	Not serious	N/A	Serious^a^	Serious^b^	Undetected	**Very Low**	↑glossiness, ↓Ra, ↑corrosion resistance
ZOE handling	1	Serious^d^	N/A	Serious^a^	Serious^b^	Undetected	**Very Low**	Improved consistency (qualitative only)

Starting certainty: Very Low for all outcomes, this is the expected baseline for *in vitro* evidence.

a Serious indirectness: laboratory findings cannot be assumed to hold in clinical settings.

b Serious imprecision: most studies used small samples, and several outcomes rested on a single study.

c Serious inconsistency: different polymer types responded in opposite directions, making a unified estimate unreliable.

d Serious risk of bias: contributing studies were rated medium or high risk on the QUIN tool.

### 2.8 Data synthesis

Due to substantial methodological and material heterogeneity across the included studies-encompassing nine distinct categories of dental material, irradiation doses spanning 1–200 kGy, acceleration energies from 25 kV to 10 MeV, and diverse outcome measures quantitative meta-analysis was not feasible. Therefore, a qualitative narrative synthesis was performed and reported in line with the Synthesis Without Meta-analysis (SWIM) guideline (28).

Studies were grouped by outcome type, and the direction, magnitude, and consistency of effects across studies are described with explicit evidence attribution for each study; where appropriate, synthesis used vote-counting based on the direction of effect. Where multiple studies assessed the same outcome, consistency across studies is noted. Dose-response patterns are reported only where directly supported by data from the included studies, with clear identification of which specific studies contribute to each observation. The synthesis of results by outcome is summarised in Tables 5a–5k.

**Table 5a:** Synthesis of results by outcome (Synthesis Without Meta-analysis, SWiM): Flexural strength.

Study (Ref.)	Material	Dose	Result vs. non-irradiated control	Direction
Hegde 2014 (7)	Filtek Z350 XT	1 kGy	170.89 → 269.06 MPa	↑ Improved
Hegde 2014 (7)	Filtek Z350 XT	3 kGy	→ 326.53 MPa	↑ Improved
Hegde 2014 (7)	Filtek Z350 XT	5 kGy	→ 377.64 MPa (*p* < 0.001)	↑ Improved
Hegde 2014 (7)	Ceram X-duo	5 kGy	120.14 → 229.33 MPa (*p* < 0.001)	↑ Improved
Hegde 2016 (8)	Filtek Z350 XT	10 kGy	172.05 → 388.60 MPa (*p* < 0.001)	↑ Improved
Hegde 2016 (8)	Filtek Bulk Fill	10 kGy	155.26 → 311.85 MPa (*p* < 0.001)	↑ Improved
Ito 2014 (15)	Self-cure acrylic (PMMA)	50 kGy	61.5 → 73.0 MPa (*p* < 0.01); optimal at 50 kGy	↑ Improved
Alshamrani 2020 (16)	3Y-PSZ zirconia (low-translucency)	70 Gy	712.45 → 588.40 MPa (*p* < 0.05)	↓ Reduced
Alshamrani 2020 (16)	5Y-PSZ zirconia (high-translucency)	70 Gy	No significant change (*p* = 0.856)	No change

Flexural strength: kGy, kilogray; Gy, gray; MPa, megapascal.

Findings are grouped under each outcome (Tables 5a–5k), with one row per material and per dose where dose-specific data were reported. Arrows denote the direction of change relative to the non-irradiated control: ↑ increase, ↓ decrease. For wear resistance and monomer leaching, a decrease is the beneficial direction. Certainty of evidence was very low (modified GRADE) for every outcome and is therefore not repeated per row (see Section 2.7).

**Table 5b:** Synthesis of results by outcome (Synthesis Without Meta-analysis, SWiM): Fracture toughness (K1c) and fracture resistance.

Study (Ref.)	Material	Dose	Result vs. non-irradiated control	Direction
Behr 2005 (5)	5 veneering composites	25–200 kGy	K1c ↑ significant (*p* < 0.05); simpler-cure systems needed higher doses	↑ Improved
Behr 2006 (11)	Stick/Sinfony FRC	100 kGy	362 → 520 N after TCML (*p* < 0.05)	↑ Improved
Behr 2006 (11)	Vectris/Targis FRC	100 kGy	383 → 575 N (*p* = 0.03); no facing fractures	↑ Improved
Kolbeck 2007 (10)	FibreKor FRC	15 kGy	74 → 134 N (*p* = 0.001)	↑ Improved
Kolbeck 2007 (10)	FibreKor FRC	33 kGy	→ 153 N (*p* = 0.001)	↑ Improved
Kolbeck 2007 (10)	FibreKor FRC	100 kGy	→ 231 N (*p* = 0.001)	↑ Improved
Faltermeier 2007 (12)	Denture base blends	25 kGy	K1c and work of fracture ↑	↑ Improved
Faltermeier 2007 (12)	Denture base blends	200 kGy	Significant deterioration (*p* < 0.005)	↓ Reduced
Faltermeier 2010 (13)	Polycarbonate (PC)	100 kGy	K1c ↑ (*p* = 0.005)	↑ Improved
Faltermeier 2010 (13)	Polyurethane (PU)	100 kGy	K1c ↑ (*p* = 0.005)	↑ Improved
Faltermeier 2010 (13)	Polyoxymethylene (POM)	100 kGy	K1c ↓ (*p* = 0.047)	↓ Reduced
Vaishnavi 2010 (9)	Adoro indirect composite	1 kGy	K1c 0.064 → 0.102 MPa·m½ (*p* < 0.05); comparable to inlay system	↑ Improved

Fracture toughness and fracture resistance: K1c, fracture toughness; FRC, fibre-reinforced composite; N, newton; kGy, kilogray; TCML, thermocycling and mechanical loading.

Findings are grouped under each outcome (Tables 5a–5k), with one row per material and per dose where dose-specific data were reported. Arrows denote the direction of change relative to the non-irradiated control: ↑ increase, ↓ decrease. For wear resistance and monomer leaching, a decrease is the beneficial direction. Certainty of evidence was very low (modified GRADE) for every outcome and is therefore not repeated per row (see Section 2.7).

**Table 5c:** Synthesis of results by outcome (Synthesis Without Meta-analysis, SWiM): Vickers hardness.

Study (Ref.)	Material	Dose	Result vs. non-irradiated control	Direction
Behr 2005 (5)	Veneering composites (ArtGlass, Sinfony)	100–200 kGy	VH ↑ (highest at 100–200 kGy)	↑ Improved
Faltermeier 2007 (12)	MMA/BDDMA denture blends	25 kGy	VH ↑ (not consistent across all blends)	↑ Improved
Faltermeier 2010 (13)	Polycarbonate (PC)	100 kGy	VH ↑ (*p* = 0.022)	↑ Improved
Faltermeier 2010 (13)	Polyurethane (PU)	100 kGy	VH ↑ (*p* = 0.032)	↑ Improved
Faltermeier 2012 (14)	Aesthetik-Line (PC)/Envision (PU)/Exper 1 & 2	100 kGy	VH ↑ (all *p* ≤ 0.028)	↑ Improved
Faltermeier 2012 (14)	Brillant (POM)	100 kGy	22.17 → 18.47 (*p* = 0.005)	↓ Reduced

Vickers hardne*s*s: VH, Vickers hardness; kGy, kilogray.

Findings are grouped under each outcome (Tables 5a–5k), with one row per material and per dose where dose-specific data were reported. Arrows denote the direction of change relative to the non-irradiated control: ↑ increase, ↓ decrease. For wear resistance and monomer leaching, a decrease is the beneficial direction. Certainty of evidence was very low (modified GRADE) for every outcome and is therefore not repeated per row (see Section 2.7).

**Table 5d:** Synthesis of results by outcome (Synthesis Without Meta-analysis, SWiM): Wear resistance.

Study (Ref.)	Material	Dose	Result vs. non-irradiated control	Direction
Behr 2005 (5)	5 veneering composites	100 kGy	Three-medium wear ↓ (*p* < 0.05)	↓ Wear (improved)
Faltermeier 2010 (13)	Polyurethane (PU)	100 kGy	Wear ↓ (*p* = 0.007); POM/PC non-significant	↓ Wear (improved)
Vaishnavi 2010 (9)	Adoro indirect composite	1 kGy	Wear scar 1.114 → 0.805 mm (*p* < 0.05)	↓ Wear (improved)

Wear resistance: kGy, kilogray; mm, millimetre.

Findings are grouped under each outcome (Tables 5a–5k), with one row per material and per dose where dose-specific data were reported. Arrows denote the direction of change relative to the non-irradiated control: ↑ increase, ↓ decrease. For wear resistance and monomer leaching, a decrease is the beneficial direction. Certainty of evidence was very low (modified GRADE) for every outcome and is therefore not repeated per row (see Section 2.7).

**Table 5e:** Synthesis of results by outcome (Synthesis Without Meta-analysis, SWiM): Bond strength.

Study (Ref.)	Material	Dose	Result vs. non-irradiated control	Direction
Behr 2005 (6)	Titanium + Metal Primer II (thiol-phosphate)	100 kGy	Shear bond 18.3 → 31.9 MPa (*p* < 0.001); stable after 12,000 thermocycles	↑ Improved
Behr 2005 (6)	CoCr + Metal Primer II	100 kGy	Tensile 18.9 → 40.8 MPa (*p* < 0.001)	↑ Improved
Behr 2005 (6)	SR-Link/Rocatec systems	100 kGy	Limited benefit	↔ Limited
Kolbeck 2007 (10)	FibreKor + SR-Link	15 kGy	Shear bond strength ↑ (*p* = 0.038)	↑ Improved
Kolbeck 2007 (10)	FibreKor + Metal Primer II	33 kGy	Shear bond strength ↑ (*p* = 0.015)	↑ Improved

Bond strength: kGy, kilogray; MPa, megapascal; CoCr, cobalt-chromium.

Findings are grouped under each outcome (Tables 5a–5k), with one row per material and per dose where dose-specific data were reported. Arrows denote the direction of change relative to the non-irradiated control: ↑ increase, ↓ decrease. For wear resistance and monomer leaching, a decrease is the beneficial direction. Certainty of evidence was very low (modified GRADE) for every outcome and is therefore not repeated per row (see Section 2.7).

**Table 5f:** Synthesis of results by outcome (Synthesis Without Meta-analysis, SWiM): Colour stability (Δ*E*).

Study (Ref.)	Material	Dose	Result vs. non-irradiated control	Direction
Behr 2005 (5)	5 veneering composites	25/100/200 kGy	*Δ*E > 3 at all doses	↑ *Δ*E (adverse)
Faltermeier 2007 (12)	Denture base blends	25 & 200 kGy	*Δ*E > 3 for all blends	↑ *Δ*E (adverse)
Faltermeier 2010 (13)	PC/PU/POM brackets	100 kGy	Visible discolouration (*Δ*E not measured)	↑ *Δ*E (adverse)
Faltermeier 2012 (14)	Aesthetik-Line bracket	100 kGy	*Δ*E 7.30 (highest)	↑ *Δ*E (adverse)
Faltermeier 2012 (14)	Exper 2 bracket (40% SiO2)	100 kGy	*Δ*E 2.95 (only acceptable value)	Acceptable

Colour stability: Δ*E*, colour difference; kGy, kilogray; SiO₂, silica; PC, polycarbonate; PU, polyurethane; POM, polyoxymethylene.

Findings are grouped under each outcome (Tables 5a–5k), with one row per material and per dose where dose-specific data were reported. Arrows denote the direction of change relative to the non-irradiated control: ↑ increase, ↓ decrease. For wear resistance and monomer leaching, a decrease is the beneficial direction. Certainty of evidence was very low (modified GRADE) for every outcome and is therefore not repeated per row (see Section 2.7).

**Table 5g:** Synthesis of results by outcome (Synthesis Without Meta-analysis, SWiM): Antimicrobial activity and biocompatibility.

Study (Ref.)	Material	Dose	Result vs. non-irradiated control	Direction
Shetty 2014 (19)	1.25% NaOCl vs. E. faecalis	1 kGy	13.75 → 16.00 mm (*p* < 0.001)	↑ Improved
Shetty 2014 (19)	1% CHX vs. E. faecalis	1 kGy	14.50 → 22.00 mm (*p* < 0.001)	↑ Improved
Shetty 2014 (19)	1.25% NaOCl vs. S. aureus	1 kGy	13.00 → 25.00 mm (*p* < 0.001)	↑ Improved
Shetty 2014 (19)	1% CHX vs. S. aureus	1 kGy	19.00 → 26.00 mm (*p* < 0.001)	↑ Improved
Shetty 2014 (19)	NaOCl/CHX (MTT; pH, colour)	1 kGy	Biocompatibility and physicochemical properties unchanged	No change

Antimicrobial activity and biocompatibility: kGy, kilogray; NaOCl, sodium hypochlorite; CHX, chlorhexidine; mm, millimetre.

Findings are grouped under each outcome (Tables 5a–5k), with one row per material and per dose where dose-specific data were reported. Arrows denote the direction of change relative to the non-irradiated control: ↑ increase, ↓ decrease. For wear resistance and monomer leaching, a decrease is the beneficial direction. Certainty of evidence was very low (modified GRADE) for every outcome and is therefore not repeated per row (see Section 2.7).

**Table 5h:** Synthesis of results by outcome (Synthesis Without Meta-analysis, SWiM): Genotoxicity and cytotoxicity.

Study (Ref.)	Material	Dose	Result vs. non-irradiated control	Direction
Hegde 2014 (18)	GIC Type IX	10 kGy	Comet tail length 24.70 → 50.82 px (*p* < 0.0001); OTM 15.39 → 26.51 (*p* = 0.0203)	↑ DNA damage (adverse)
Ravi 2014 (29)	Unite adhesive	2 kGy	%DNA-in-tail 8.23 → 21.66; haemolysis non-significant	↑ DNA damage (adverse)
Ravi 2014 (29)	MIP adhesive	2 kGy	%DNA-in-tail 7.86 → 31.17; haemolysis ↑	↑ Damage (adverse)
Ravi 2014 (29)	GC Fuji adhesive	2 kGy	%DNA-in-tail 8.56 → 15.85; haemolysis ↑	↑ Damage (adverse)

Genotoxicity and cytotoxicity: kGy, kilogray; GIC, glass-ionomer cement.

Findings are grouped under each outcome (Tables 5a–5k), with one row per material and per dose where dose-specific data were reported. Arrows denote the direction of change relative to the non-irradiated control: ↑ increase, ↓ decrease. For wear resistance and monomer leaching, a decrease is the beneficial direction. Certainty of evidence was very low (modified GRADE) for every outcome and is therefore not repeated per row (see Section 2.7).

**Table 5i:** Synthesis of results by outcome (Synthesis Without Meta-analysis, SWiM): Monomer leaching.

Study (Ref.)	Material	Dose	Result vs. non-irradiated control	Direction
Sajnani 2014 (21)	Filtek Z350 XT Restorative	1–5 kGy	Lowest leaching at 3 kGy; non-irradiated highest; rises again at 5 kGy	↓ Leaching (improved)
Sajnani 2014 (21)	Filtek Z350 XT Flowable	1–5 kGy	Lowest leaching at 3 kGy; BisGMA > TEGDMA	↓ Leaching (improved)

Monomer leaching: kGy, kilogray; BisGMA, bisphenol A-glycidyl methacrylate; TEGDMA, triethylene glycol dimethacrylate.

Findings are grouped under each outcome (Tables 5a–5k), with one row per material and per dose where dose-specific data were reported. Arrows denote the direction of change relative to the non-irradiated control: ↑ increase, ↓ decrease. For wear resistance and monomer leaching, a decrease is the beneficial direction. Certainty of evidence was very low (modified GRADE) for every outcome and is therefore not repeated per row (see Section 2.7).

**Table 5j:** Synthesis of results by outcome (Synthesis Without Meta-analysis, SWiM): Surface polishing of cast titanium.

Study (Ref.)	Material	Dose	Result vs. non-irradiated control	Direction
Tokunaga 2009 (17)	Cast titanium	20 pulses (5.0 J/cm^2^)	Glossiness 4.1 → 154 Gs; Ra 2.34 → 0.31 µm	↑ Finish improved
Tokunaga 2009 (17)	Cast titanium	20–80 pulses	Corrosion resistance improved (resting potential ↑, current density ↓)	↑ Improved

Surface polishing of cast titanium: Gs, gloss units; Ra, arithmetic mean surface roughness; µm, micrometre; J/cm^2^, joules per square centimetre.

Findings are grouped under each outcome (Tables 5a–5k), with one row per material and per dose where dose-specific data were reported. Arrows denote the direction of change relative to the non-irradiated control: ↑ increase, ↓ decrease. For wear resistance and monomer leaching, a decrease is the beneficial direction. Certainty of evidence was very low (modified GRADE) for every outcome and is therefore not repeated per row (see Section 2.7).

**Table 5k:** Synthesis of results by outcome (Synthesis Without Meta-analysis, SWiM): Handling properties of ZOE.

Study (Ref.)	Material	Dose	Result vs. non-irradiated control	Direction
Hegde 2013 (20)	ZOE root canal sealer	10 kGy	Dry/crumbly → thicker, paste-like consistency (qualitative)	Altered

Handling properties of ZOE: ZOE, zinc oxide eugenol; kGy, kilogray.

Findings are grouped under each outcome (Tables 5a–5k), with one row per material and per dose where dose-specific data were reported. Arrows denote the direction of change relative to the non-irradiated control: ↑ increase, ↓ decrease. For wear resistance and monomer leaching, a decrease is the beneficial direction. Certainty of evidence was very low (modified GRADE) for every outcome and is therefore not repeated per row (see Section 2.7).

## 3 Results

### 3.1 Study selection

A total of 897 records were identified through database searching: PubMed/MEDLINE (*n* = 145) Scopus (*n* = 419), and Web of Science (*n* = 333). After removal of 524 duplicate records, 373 unique records were screened by title and abstract, of which 331 were excluded. The remaining 42 reports were retrieved and assessed in full text, of which 29 were excluded with reasons—the material studied was not a dental material (*n* = 13) the intervention was not electron beam irradiation (*n* = 8) the study was conducted *in vivo* or in animals (*n* = 3); electron beam irradiation was not used to modify a dental material, for example for sterilisation or synthesis (*n* = 4) and the study did not assess post-irradiation properties of the dental material (*n* = 1). A detailed list of all 29 excluded studies with individual reasons is provided in Supplementary Table S3. Thirteen studies were included from the database search. A further five studies were identified through reference-list (hand) searching (Studies 4, 12, 13, 14, and 17) and met all eligibility criteria. In total, eighteen studies were included in the qualitative synthesis. The study selection process is illustrated in the PRISMA 2020 flow diagram (Figure 1).

### 3.2 Study characteristics

Eighteen studies published between 2005 and 2020 met the inclusion criteria (Table 1). All employed an *in vitro* experimental design. The studies originated from Germany (*n* = 7) (5, 6, 10–14), India (*n* = 6) (7, 8, 18–21), Japan (*n* = 2) (15, 17), and Canada (*n* = 1) (16). The dental materials investigated included: veneering composites (*n* = 1) (5), nanocomposites (*n* = 3) (7, 8, 21), FRCs (*n* = 2) (10, 11), experimental denture base polymers (*n* = 1) (12), orthodontic bracket polymers (*n* = 2) (13, 14), alloy-to-resin bonding systems (*n* = 1) (6), monolithic zirconia (*n* = 1) (16), self-curing acrylic resin (*n* = 1) (15), indirect composites (*n* = 1) (9), GIC (*n* = 1) (18), dental irrigants (*n* = 1) (19), cast titanium (*n* = 1) (17), and ZOE (*n* = 1) (20).

EB irradiation doses ranged from 1 kGy to 200 kGy. Acceleration energies ranged from 110 kV (15) to 10 MeV (5–8, 10, 11, 13, 14). One study used a medical linear accelerator at 6 MV with 70 Gy (16). Seven studies (5, 6, 10–14) used the Rhodotron electron accelerator (BGS Beta Gamma Service, Germany), which was the most common equipment. Another seven studies (7, 8, 18–21) used the Microtron Centre at Mangalore University, India. Critical irradiation parameters such as dose rate, number of passes, and ambient atmosphere were inconsistently reported: dose rate was specified in only 2 studies (6, 11), and atmosphere (vacuum, air, or inert) was explicitly stated in only 1 study (13).

These three parameters are not incidental descriptors but mechanistic determinants of how electron-beam irradiation acts on polymeric dental materials, so their inconsistent reporting bears directly on the robustness of our findings. Dose rate governs the instantaneous radical concentration and the rate at which dissolved oxygen is consumed relative to its diffusion into the specimen: at high dose rates the irradiated zone becomes effectively oxygen-depleted, favouring radical recombination and cross-linking-the route to the mechanical gains we observed whereas lower dose rates permit continuous re-oxygenation and shift the balance towards oxidative chain scission and degradation. The number of passes modifies both the thermal history of the specimen, since single high-dose passes generate greater heat accumulation with the attendant risk of exceeding the glass-transition temperature, and the opportunity for oxygen to re-diffuse between passes. Ambient atmosphere acts in the same direction: identical nominal doses delivered in air vs. an inert nitrogen or argon atmosphere can produce opposite results oxidative degradation and discolouration in the former, predominantly cross-linking in the latter.

Because these moderators were reported inconsistently, the nominal absorbed dose (kGy) is an incomplete description of the exposure, and any dose-response relationship inferred from it is necessarily confounded. We cannot determine whether the divergent outcomes seen at comparable nominal doses—mechanical Improvement in some studies, degradation or discolouration in others-reflect genuine differences between materials or merely unreported differences in dose rate, fractionation, or atmosphere. This weakens the review's robustness in three concrete ways: it prevents us from locating, with any precision, the dose thresholds at which effects turn from beneficial to harmful; it accounts for part of the unexplained heterogeneity that, together with the *in vitro* design, underpins the very-low GRADE certainty rating; and it undermines reproducibility, since a protocol specified by total dose alone cannot be reliably repeated. The dose-dependent patterns reported here should therefore be read as associations with nominal dose rather than validated dose-response relationships, and future studies should report, as a minimum, absorbed dose, dose rate, beam energy, number of passes, irradiation atmosphere, and specimen temperature.

### 3.3 Synthesis of results by outcome

The synthesis of results by outcome is summarised in Tables 5a–5k; the text below describes each outcome in detail.

#### 3.3.1 Flexural strength

Four studies evaluated flexural strength (Tables 5a) (7, 8, 15, 16). Hegde et al. (7) reported that the flexural strength of Filtek Z350 XT increased from 170.89 ± 9.07 MPa (control) to 269.06 ± 94.91 MPa at 1 kGy, 326.53 ± 54.74 MPa at 3 kGy, and 377.64 ± 74.5 MPa at 5 kGy (*p* < 0.001). The flexural strength of Ceram X duo increased from 120.14 ± 7.12 MPa to 229.33 ± 59.15 MPa at 5 kGy (*p* < 0.001). Hegde et al. (8) confirmed these findings with Filtek Z350 XT (control 172.05 ± 2.93 MPa; 10 kGy 388.60 ± 11.13 MPa, *p* < 0.001) and Filtek Bulk Fill (control 155.26 ± 2.54 MPa; 10 kGy 311.85 ± 4.96 MPa, *p* < 0.001). Ito et al. (15) demonstrated a novel approach of irradiating polymer powder before mixing, achieving maximum flexural strength at 50 kGy (73.0 ± 1.9 MPa vs. 61.5 ± 3.0 MPa; *p* < 0.01), with ESR spectroscopy confirming increased free radical generation and molecular weight measurements showing decreased molecular weight consistent with chain scission followed by re-crosslinking during polymerization. Alshamrani and De Souza (16) found that 70 Gy ionising radiation decreased flexural strength of low-translucency 3Y-PSZ zirconia from 712.45 ± 77.04 MPa to 588.40 ± 143.82 MPa (*p* < 0.05), though high-translucency 5Y-PSZ zirconia was unaffected (*p* = 0.856). This contrasting result for zirconia is consistent with its ceramic (non-polymeric) nature, where radiation-induced tetragonal-to-cubic phase transformation (∼20 wt% by XRD) rather than cross-linking governs the property change (16).

#### 3.3.2 Fracture toughness and fracture resistance

Five studies assessed fracture toughness or resistance (Table 5b) (5, 9–13). Behr et al. (5) demonstrated significant K1c increases across five veneering composites at various doses (*p* < 0.05), with composites having simpler curing processes (light-cured only) requiring higher doses (100–200 kGy) for optimal improvement, while more complex systems (heat- and light-cured under N_2_ atmosphere) showed maximal benefit at 25 kGy. Behr et al. (11) showed that irradiated Stick/Sinfony FRC achieved fracture resistance of 520 ± 31 N vs. 362 ± 41 N after TCML (*p* < 0.05), and irradiated Vectris/Targis achieved 575 ± 57 N vs. 383 ± 51 N (*p* = 0.03). Notably, two of eight non-irradiated Vectris/Targis specimens suffered facing fractures during TCML, while no irradiated specimens failed (11). Kolbeck et al. (10) found that FibreKor fracture strength increased from 74 N (control) to 134 N (15 kGy), 153 N (33 kGy), and 231 N (100 kGy) (*p* = 0.001 at each step). Faltermeier et al. (12) reported that 25 kGy improved K1c and work of fracture for most experimental denture base blends, but 200 kGy caused significant deterioration (*p* < 0.005), consistent with excessive chain scission at high doses. Faltermeier et al. (13) found polycarbonate (K1c, *p* = 0.005) and polyurethane (*p* = 0.005) bracket materials benefited from 100 kGy irradiation, while polyoxymethylene showed decreased K1c (*p* = 0.047).

Vaishnavi et al. (9) found EB-irradiated indirect composite achieved K1c values comparable to the manufacturer's inlay system (0.102 ± 0.002 MPa m½) vs. 0.112 ± 0.002 MPa m½) and superior to autoclave-cured (0.080 ± 0.001 MPa m½) and control specimens (0.064 ± 0.002 MPa m½; *p* < 0.05).

#### 3.3.3 Vickers hardness

Five studies evaluated VH (Table 5c) (5, 8, 12, 13). Behr et al. (5) reported significant VH increases for most composites, with ArtGlass and Sinfony showing highest values at 100–200 kGy. Faltermeier et al. (14) found VH improved for Aesthetik-Line (PC, *p* = 0.028), Envision (PU, *p* = 0.005), and both UDMA-based experimental brackets (Exper 1 *p* = 0.005; Exper 2 *p* = 0.005) after 100 kGy, but Brillant (POM) showed significantly decreased VH (22.17 ± 1.43 to 18.47 ± 0.73; *p* = 0.005) (14). Faltermeier et al. (12) observed VH increases at 25 kGy for MMA/BDDMA blends but not consistently across all formulations. Faltermeier et al. (13) confirmed VH improvements for PC (*p* = 0.022) and PU (*p* = 0.032) but not POM.

#### 3.3.4 Wear resistance

Three studies evaluated wear resistance (Table 5d) (5, 9, 13). Behr et al. (5) demonstrated that all five veneering composites showed significantly reduced three-medium wear after 100 kGy (*p* < 0.05), with Targis, Solidex, and Sinfony showing the greatest improvements. Faltermeier et al. (13) found significantly improved wear resistance for PU bracket material (*p* = 0.007) but non-significant changes for POM and PC. Vaishnavi et al. (9) reported lower wear scar diameters for EB-irradiated specimens (0.805 ± 0.004 mm) compared to autoclave-cured (0.968 ± 0.004) and control (1.114 ± 0.070 mm; *p* < 0.05).

#### 3.3.5 Bond strength

Two studies assessed bond strength (Table 5e) (6, 10). Behr et al. (6) investigated three primer systems on titanium and CoCr alloys. Thiol-phosphate primers (Metal Primer II) combined with 100 kGy EB produced SBS of 31.9 ± 6.1 MPa on titanium vs. 18.3 ± 3.1 MPa control (*p* < 0.001); and TS of 40.8 ± 2.2 MPa on CoCr vs. 18.9 ± 1.2 MPa control (*p* < 0.001). After 12,000 thermal cycles, bond strength remained at 37.6 ± 4.9 MPa (SBS) and 38.7 ± 1.8 MPa (TS), demonstrating durability (6). Phosphate ester (SR Link) and silicoating (Rocatec) systems showed limited benefit (6). Kolbeck et al. (10) found SBS improvements for FibreKor with SR-Link at 15 kGy (*p* = 0.038), Metal Primer II at 33 kGy (*p* = 0.015), and Clearfil SE Bond at 33 kGy (*p* = 0.015), but effects were dependent on the FRC-pretreatment combination.

#### 3.3.6 Colour stability

Four studies evaluated colour changes (Table 5f) (5, 12–14). Three of these studies reported *Δ*E values exceeding the clinically acceptable threshold of 3.3 for nearly all material-dose combinations, while one reported visible discolouration without direct *Δ*E measurement. Behr et al. (5) reported *Δ*E > 3 for all five composites at all doses (25, 100, 200 kGy). Faltermeier et al. (12) found *Δ*E > 3 for all six denture base blends at both 25 and 200 kGy. Faltermeier et al. (14) reported Aesthetik-Line brackets had the highest ΔE (7.30) and Exper 2 brackets (40 vol% SiO2 filler) had the lowest ΔE (2.95), which was the only clinically acceptable value reported across all four studies. Faltermeier et al. (13) did not directly measure colour but reported visible discolouration. The consistent finding across all colour-measuring studies is that EB irradiation produces clinically unacceptable discolouration in most dental polymers, with the possible exception of highly filled composites.

#### 3.3.7 Antimicrobial activity and biocompatibility

One study (19) evaluated EB irradiation of dental irrigants (Table 5g) (NaOCl 1.25%, 2.5%; CHX 1%, 2%). Shetty et al. (19) reported that 1 kGy significantly increased antimicrobial activity of 1.25% NaOCl and 1% CHX against E. faecalis (16 ± 0.0 mm vs. 13.75 ± 0.95 mm control) and S. aureus (25 ± 0.0 mm vs. 13 ± 0.81 mm), without altering biocompatibility (MTT assay) or physicochemical properties (pH, colour, sedimentation) Enhanced activity was maintained after 180 days storage at 4 °C. It should be noted that the mechanism governing EB effects on aqueous irrigant solutions, likely involving radical-mediated oxidative species generation in solution, is fundamentally different from the cross-linking/scission mechanism in solid polymers.

#### 3.3.8 Genotoxicity

Two studies assessed genotoxicity (Table 5h). Hegde et al. (18) found that GIC Type IX, irradiated at 10 kGy caused significantly increased DNA damage in human lymphocytes, including tail length 50.82 ± 20.4 vs. 24.70 ± 24.70 px (*p* < 0.0001) and olive moment 26.51 ± 9.92 vs. 15.39 ± 4.67 (*p* = 0.0203) by comet assay, and increased DNA diffusion (125.5 ± 17.94 vs. 103.31 ± 14.75; *p* < 0.0001) (18). The authors attributed this finding to the release of unbound polyacrylic acid and fluoride ions due to chain breakage (18). This finding is important because it demonstrates that EB irradiation does not universally improve dental material biocompatibility and highlights the need for material-specific biocompatibility evaluations. A second study (Ravi et al.) (29) evaluating three orthodontic bonding adhesives reported a similar adverse effect: after 2 kGy irradiation, the percentage of DNA in the comet tail increased for all three adhesives, with increased olive tail moment and increased haemolysis in two of the three materials.

#### 3.3.9 Monomer leaching

One study (21) evaluated monomer release (Table 5i). Sajnani et al. (21) measured BisGMA and TEGDMA leaching from Filtek Z350 XT Restorative and Flowable, by HPLC. At 24 h, minimal leaching occurred at 3 kGy for both materials, while non-irradiated samples showed maximal leaching. At 5 kGy, leaching increased, which suggests chain scission at higher doses. BisGMA release exceeded TEGDMA, likely because 100% ethanol (the storage medium) has a solubility parameter similar to BisGMA. After 7 days, leaching generally decreased, except for TEGDMA from restorative composites (21).

#### 3.3.10 Surface polishing of cast Titanium

One study (17) investigated large-area EB for titanium polishing (Table 5j). Tokunaga et al. (17) reported that after 20 pulses of EB irradiation (5.0 J/cm^2^, 25 kV), surface glossiness increased from 4.1 to 154 Gs (hand-polished: 72 Gs), and Ra decreased from 2.34 to 0.31 µm. Corrosion resistance improved, as evidenced by increased resting potential and decreased current density in anodic polarisation. Cross-sectional SEM revealed a smooth resolidified layer approximately 7 µm deep while the bulk dendritic cast structure remained unchanged. The mechanism is surface melting and resolidification, facilitated by titanium's low thermal conductivity (21.9 W/m·K) (17).

#### 3.3.11 Handling properties of ZOE

One study (20) assessed ZOE manipulation (Table 5k). Hegde et al. reported that EB irradiation at 10 kGy improved the consistency of ZOE sealer from a dry, crumbly mixture to a thicker, more paste-like consistency suitable for clinical application (20). This was a qualitative observational study without statistical analysis and represents the lowest level of evidence among the included studies.

### 3.4 Risk of bias assessment

**Figure 2:**
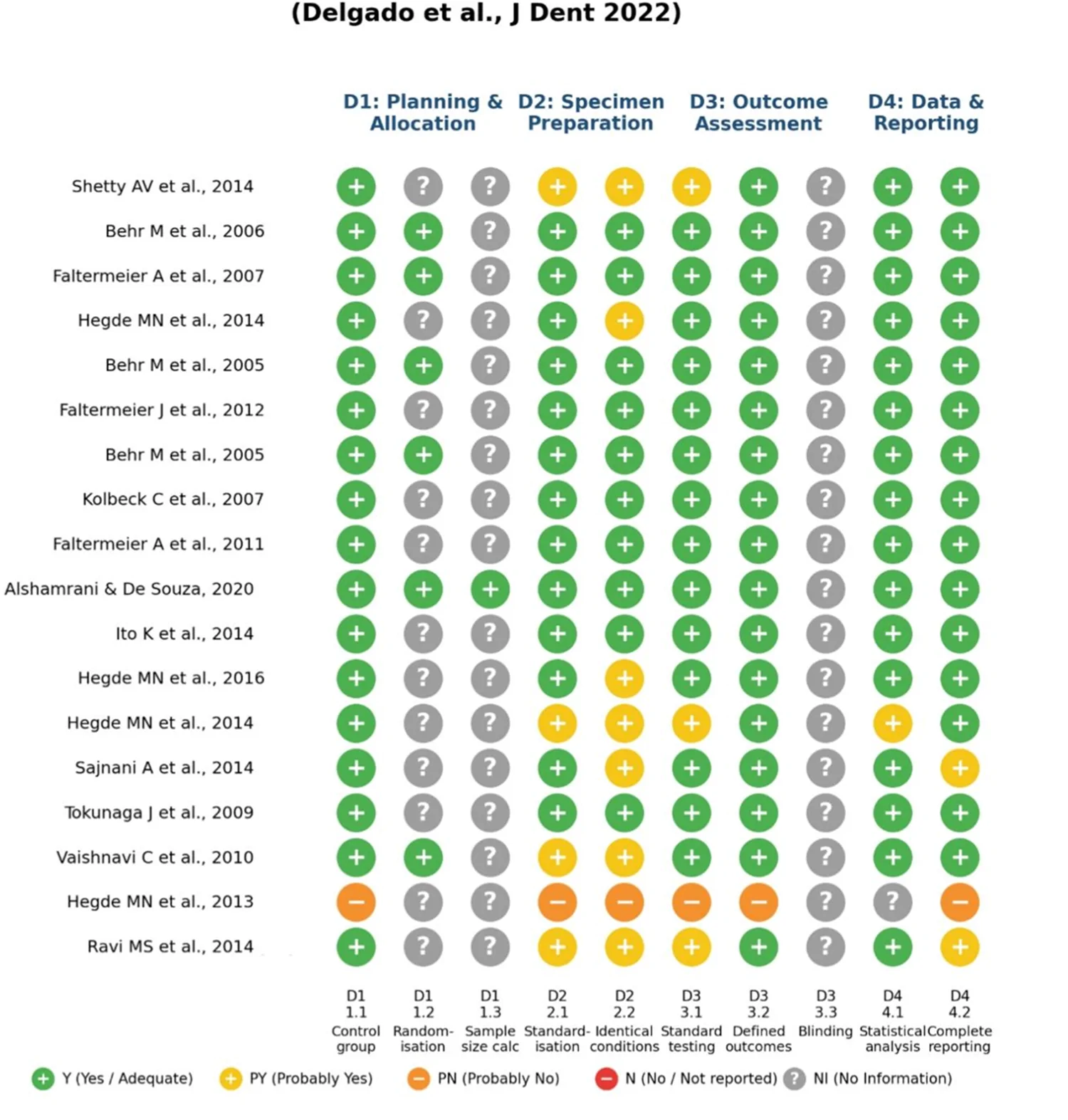
RoBDEMAT risk of bias traffic light plot for included studies (n = 18). Domains: D1 = Planning and allocation; D2 = Specimen preparation; D3 = Outcome assessment; D4 = Data treatment and reporting. Judgements: Y = Yes (green); PY = Probably Yes (amber); PN = Probably No (orange); N = No (red); NI = No Information (grey).

The RoBDEMAT assessment (Table 2, Figure 2) revealed that all 18 studies included a control or comparison group (Item 1.1). Randomisation of specimens (Item 1.2) was explicitly reported in 6 of 18 studies (35%) (5, 6, 9, 11, 12, 16). Sample size calculation (Item 1.3) was reported in only 1 study (6%) (16). Standardisation of specimen preparation following ISO standards or manufacturer instructions (Item 2.1) was adequate in 14 studies. Identical experimental conditions (Item 2.2) were adequately described in 12 studies. Standardised testing methods (Item 3.1) were used in 15 studies. Blinding of outcome assessors (Item 3.3) was not reported in any of the 18 studies, a recognized limitation of *in vitro* mechanical testing where instruments operate independently of operator's awareness of group allocation (23). Statistical analysis (Item 4.1) was appropriate in 16 of 18 studies, with Hegde et al. (20) being the exception (qualitative assessment only).

**Figure 3:**
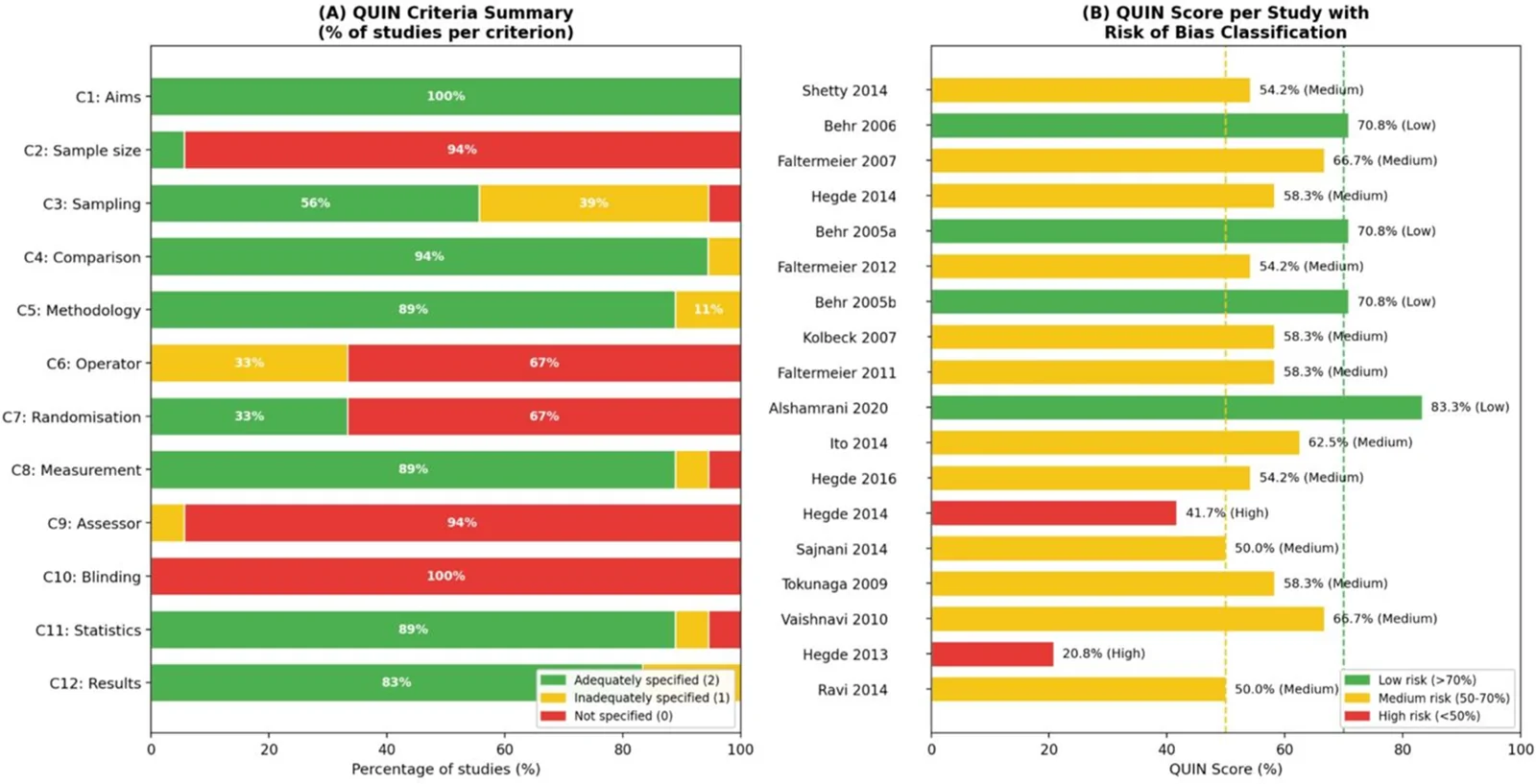
QUIN quality assessment summary. (A) Percentage of studies meeting each of the 12 QUIN criteria. (B) Individual study QUIN scores with risk of bias classification (>70% = Low; 50–70% = Medium; < 50% = High).

**The QUIN quality assessment** (Table 3, Figure 3) classified 4 studies (22%) as low risk (5, 6, 11, 16), 12 (67%) as medium risk (7–10, 12–15, 17, 19, 21, 29), and 2(11%) as high risk of bias (18, 20).

**The inter-rater agreement was *κ*** **≥** **0.78.** The most rigorous study methodologically was Alshamrani and De Souza (16), which was the only study to report sample size calculation, randomization, standardised fatigue testing (staircase method with 100,000 cycles), and multiple characterization methods (XRD, SEM, EDS).

### 3.5 Certainty of evidence

The **modified GRADE assessment (**Table 4**)** rated the certainty of evidence as “Very Low” for all 12 outcome categories. This is primarily attributable to: (a) the inherent pre-clinical nature of all evidence (starting baseline of “Very Low” for *in vitro* studies); (b) serious indirectness for all outcomes (laboratory test results on material specimens cannot be directly extrapolated to clinical performance in the oral environment); and (c) serious imprecision for outcomes assessed in only one or two studies. Risk of bias was judged as “not serious” for outcomes where the majority of contributing studies were rated as low risk by QUIN, and as “serious” where contributing studies had medium or high risk ratings. Inconsistency was judged as “serious” only for Vickers hardness, where different polymer types responded in opposite directions to EB irradiation (13). Publication bias was rated as “undetected” across all outcomes; formal assessment was not possible due to the qualitative nature of the synthesis, though the inclusion of studies reporting both beneficial and detrimental effects argues against systematic positive publication bias.

## 4 Discussion

### 4.1 Summary of main findings

This systematic review is, to the best of our knowledge, the first to comprehensively evaluate the effect of EB irradiation on dental materials using validated risk of bias tools [RoBDEMAT (23) and QUIN (24)], a formal certainty of evidence assessment [modified GRADE (25)], and PRISMA 2020-compliant methodology (22). The synthesis of 17 *in vitro* studies spanning 15 years (2005–2020) reveals a consistent pattern: EB irradiation, at low-to-moderate doses, improves the mechanical properties of dimethacrylate-based dental polymers and their composites, while producing clinically unacceptable colour changes in most tested materials. The effect is material-specific and dose-dependent, with no universally optimal irradiation protocol identifiable from the current evidence.

### 4.2 Evidence-based mechanistic interpretation

The following mechanistic discussion is anchored in observations directly reported by the included studies. Broader radiation chemistry context is provided where necessary but is explicitly identified as such.

#### 4.2.1 Cross-linking vs. chain scission: evidence from included studies

The two competing EB-induced reactions cross-linking and chain scission were directly invoked as explanatory mechanisms by the majority of included studies (5–8, 10–13, 15). The evidence for cross-linking as the dominant mechanism at low-to-moderate doses comes from several converging observations within the included studies: (a) Ito et al. (15) provided direct experimental evidence through ESR spectroscopy, demonstrating the generation of free radicals from main-chain scission in irradiated PMMA powder, with radical quantity increasing linearly up to 100 kGy, and molecular weight decreasing from 960,000 to 390,000, yet the flexural strength of the resulting polymerized material increasing consistent with re-cross-linking during subsequent monomer polymerization; (b) Faltermeier et al. (12) demonstrated the transition from beneficial to detrimental effects when dose increased from 25 to 200 kGy across all six denture base blends, consistent with the shift from predominantly cross-linking to chain scission at high doses; (c) Behr et al. (5) showed that composites with simpler curing systems required higher doses for optimal improvement, which they attributed to higher residual double-bond content providing more sites for radiation-induced cross-linking; and (d) Sajnani et al. (21) found minimum monomer leaching at 3 kGy (suggesting maximum cross-linking) but increased leaching at 5 kGy (suggesting onset of chain scission).

At the molecular level, EBI bombards polymer chains with high-energy electrons, stripping electrons and exciting atoms to produce free radicals (30, 31). At low-to-moderate doses, these radicals mainly drive intermolecular cross-linking via radical recombination. At higher doses (>100 kGy), radical accumulation tips the balance toward chain scission and oxidative degradation—peroxy and hydroxyl radicals accelerate backbone cleavage (32, 33).The crosslinking-to-scission ratio depends on dose, dose rate, and environmental conditions (10, 31).

#### 4.2.2 Material-specificity of the EB response: evidence from included studies

Across included studies, EB response was governed by polymer composition. Dimethacrylate-based systems (BisGMA, UDMA, TEGDMA) consistently benefited from EB irradiation across multiple independent studies (5–10). Faltermeier et al. (2011, 2012) found that polyoxymethylene (POM) brackets deteriorated after irradiation—K1c (13, 14) both dropped-while polycarbonate and polyurethane brackets improved. POM's highly crystalline structure likely explains this: crystalline regions are particularly vulnerable to chain scission (13, 14). PMMA-containing systems were less predictable. EverStick (BisGMA/PM*ΜΑ* matrix) peaked at 15 kGy; beyond that, scission in the PMMA component likely offset cross-linking gains in the BisGMA (10). The ceramic material (zirconia) behaved differently with irradiation triggered a tetragonal-to-cubic phase transformation, not anything resembling a polymer mechanism (16).

Material composition emerges as a critical determinant of radiation response. Methacrylate-based composites exhibit enhanced radiation stability compared with aliphatic polymers due to resonance stabilization of aromatic radicals (1). Inorganic fillers—particularly silica, zirconia, and hydroxyapatite—provide additional protection against oxidative degradation by scavenging free radicals and reducing oxygen diffusion into the polymer matrix (30).

#### 4.2.3 The colour change problem: evidence from included studies

Colour change was the most consistently reported adverse effect, documented in all four studies that measured it (5, 12–14). Behr et al. (5) attributed the discolouration to colour-centre anion defects in crystalline lattice regions with linked surplus electrons and noted that materials with the most complex curing systems (and presumably highest degree of conversion) showed the lowest colour changes (5). Faltermeier et al. (14) observed that the experimental bracket Exper 2, which had 40 vol% functional silane-treated SiO2 fillers, achieved clinically acceptable *Δ*E values, while other unfilled or lightly filled polymers exceeded the 3.3 threshold (14). This observation from the included studies suggests that a high proportion of inorganic filler may buffer the polymeric matrix against irradiation-induced discolouration. Faltermeier et al. (12) noted that discolouration appeared independent of dose (25 vs. 200 kGy produced similar *Δ*E values), suggesting that colour centres may form at relatively low thresholds. The broader radiation chemistry literature (from outside the included studies) attributes polymer yellowing to oxidation of unreacted C = C bonds producing coloured peroxide compounds (33), a mechanism also cited by Faltermeier et al. (14).

Aromatic polymers demonstrate superior colour stability compared to aliphatic variants, as conjugated systems can dissipate radical energy through resonance without chromophore formation (34).

### 4.3 Dose-response patterns: study-by-study evidence

Rather than proposing universal dose thresholds — which would be premature given the heterogeneity — the following study-specific dose-response observations are reported:

At 1–5 kGy: Two studies (7, 8) on nanocomposites (Filtek Z350 XT, Ceram X duo, Filtek Bulk Fill) showed significant dose-dependent improvements in flexural strength, with effects detectable from as low as 1 kGy. Sajnani et al. (21) found optimal monomer leaching reduction at 3 kGy. Shetty et al. (19) reported maximal antimicrobial enhancement at 1 kGy.

At 15–33 kGy: Kolbeck et al. (10) showed optimal fracture strength improvement for everStick at 15 kGy and significant SBS improvements at 15–33 kGy. Faltermeier et al. (12) achieved the best mechanical property profile at 25 kGy for denture base polymers.

At 100 kGy: Behr et al. (5, 6, 11) and Faltermeier et al. (13, 14) used 100 kGy in multiple studies with generally positive results for composites, FRCs, and alloy-to-resin bonds, but with universal colour changes. FibreKor showed continued improvement up to 100 kGy (10).

At 200 kGy: Faltermeier et al. (12) demonstrated significant deterioration of all mechanical properties across all six denture base blends, establishing 200 kGy as clearly excessive for these materials. Behr et al. (5) showed mixed results at 200 kGy depending on the composite system.

These observations suggest that the optimal dose range is material-dependent and no reliable universal threshold can currently be proposed.

### 4.4 Comparison with previous literature

To the best of our knowledge, no previous systematic review has addressed EB irradiation of dental materials specifically. The present review provides the first systematic synthesis of EB-specific evidence, which differs from gamma irradiation in dose rate, penetration depth, and the possibility of targeted application, thus addressing a distinct gap in the dental materials literature.

### 4.5 Clinical implications and practical considerations

The clinical translation of EB irradiation for dental materials faces several practical barriers. First, the colour change problem, documented in multiple colour-measuring studies (5, 14), currently limits the application to non-aesthetic components or materials with high filler content. Second, the cost and logistics of EB irradiation equipment make individual restoration irradiation economically impractical (11). The included studies suggest several feasible pathways: (a) irradiation of prefabricated mass-produced components such as artificial teeth, FRC frameworks, or orthodontic brackets, where costs can be distributed (11); (b) pre-irradiation of polymer raw materials (powder) before conventional processing, as demonstrated by Ito et al. (15), which would not require irradiation equipment at individual dental offices; and (c) thiol-phosphate primer/EB combinations for adhesive prosthodontics, where the 3–4 fold bond strength improvement (6) and thermal cycling stability (6) could justify the additional processing step for high-value restorations.

However, given the very low certainty of evidence (**modified GRADE,** Table 4) and the exclusively *in vitro* nature of the included studies, no clinical recommendations can be made at this stage. These findings should be interpreted as hypothesis-generating for future clinical investigations.

### 4.6 Strengths

This review has several methodological strengths. First, it is the first systematic review to comprehensively address EB irradiation of dental materials. Second, the review employed two risk of bias tools developed specifically for *in vitro* dental research: RoBDEMAT (23), the first domain-based RoB tool developed for preclinical dental materials research through international Delphi consensus, with endorsement from the IADR-DMG and EFCD; and the QUIN Tool (24), a quantitative scoring instrument developed by expert consensus with reported reliability testing. We note that, as recently developed instruments, neither tool has yet undergone extensive independent psychometric validation; they were selected for their direct relevance to *in vitro* dental research rather than an established validation record.

This dual approach combines a domain-level checklist (23) with quantitative low/medium/high risk classification (24), Third, evidence certainty was rated using a modified GRADE approach, with an honest starting point: all *in vitro* evidence begins at “Very Low” certainty, stated plainly rather than buried. Fourth, the discussion keeps a clear line between findings from included studies and background context drawn from elsewhere.

### 4.7 Interpretation in light of risk of bias

Across both appraisal tools the included studies shared a consistent set of methodological weaknesses that bears directly on how confidently their findings can be read. RoBDEMAT showed that, although all 18 studies used an appropriate non-irradiated control and most applied standardised specimen preparation and testing, none reported blinding of outcome assessment, randomisation of specimen allocation was described in only about a third, and an *a priori* sample-size calculation was reported in just one study. QUIN gave a concordant picture blinding not specified in any study (100%), sample-size calculation in only one (6%) (16), and operator and assessor details absent in roughly two-thirds and classified four studies (22%) as low risk (5, 6, 11, 16), twelve (67%) as medium risk (7–10, 12–15, 17, 19, 21, 29), and two (11%) as high risk of bias (18, 20). Because blinding and formal power calculations were almost universally absent, even the statistically significant effects carry a real possibility of measurement bias and of unstable, underpowered estimates; this is the principal driver of the very-low GRADE certainty assigned to every outcome.

These risks are not distributed evenly, so the main findings differ in the weight they can bear. The most reproducible signal-improved flexural strength, fracture toughness, hardness, and wear resistance in dimethacrylate-based polymers at low-to-moderate doses is supported by the largest number of studies (three to five per outcome), includes the only studies rated at low risk of bias [the three Behr studies and Alshamrani (5, 6, 11, 16)], and was measured with instrumented quantitative tests that are comparatively resistant to assessor bias; it is therefore the most defensible conclusion, although the absence of power calculations leaves the magnitude of benefit uncertain. The adverse biological signals rest on far weaker foundations: genotoxicity is informed by only two small studies (18, 20), one of which [Hegde 2014 (18), glass ionomer cement] was among the lowest-scoring in the review (41.7%, high risk), and cytotoxicity by a single study whose haemolysis assay used only two replicates and whose comet data carried very wide standard deviations (e.g., 109.42 ± 70.32 px). These should be read as hypothesis-generating indications of the direction of effect, not as reliable estimates of its size. The conclusion most vulnerable to bias is the altered handling of zinc oxide eugenol sealer, which derives entirely from the single highest-risk study (20.8%, high risk) (20). reporting a qualitative outcome without blinding, and should be regarded as preliminary.

Two further factors compound these risks. Several outcomes- antimicrobial activity, monomer leaching, cytotoxicity, zirconia behaviour, titanium surface finishing, and sealer handling were each examined in only one study, so their results could neither be corroborated nor checked for consistency. In addition, five included studies (7, 8, 18, 20, 21) share authorship with members of the review team, and two of the higher-risk studies (18, 20) fall within this group; although a pre-specified robustness check indicated that the overall conclusions did not depend on these studies, the convergence of higher bias with author overlap warrants particular caution when interpreting the biological outcomes. Taken together, the risk-of-bias analysis tempers the review's conclusions: the evidence supports a provisional, direction-of-effect statement that low-to-moderate doses can enhance the mechanical properties of polymer-based dental materials while higher doses risk discolouration and biological harm, but it does not support quantitative dose recommendations, and all conclusions remain at very low certainty pending blinded, adequately powered, and independently conducted studies.

### 4.8 Limitations

The following limitations should be considered, distinguishing limitations of the included primary studies from limitations of the review itself. Several of these limitations relate to the methodological risks discussed in Section 4.7; they are listed here for completeness.

First, all 18 included studies were conducted *in vitro*, and *in vitro* findings don't translate directly to clinical performance. Second, the studies also varied considerably across materials, irradiation parameters, and outcome measures-too much variation to allow for a meta-analysis.Third, key irradiation parameters-dose rate, number of passes, and ambient atmosphere were inconsistently reported across the included studies. As discussed in the Study Characteristics section, these parameters mechanistically determine whether irradiation favours cross-linking or oxidative degradation, so their omission means nominal absorbed dose (kGy) only partially describes the exposure. The dose-dependent patterns reported here should therefore be interpreted as associations with nominal dose rather than validated dose-response relationships.

Fourth, none of the studies reported blinding—though this is a known limitation of *in vitro* mechanical testing and often hard to avoid (23). Fifth, only one study reported a sample size calculation (16). Sixth, several outcomes [antimicrobial activity (19), monomer leaching (21), surface polishing (17), handling properties (20)] were assessed in only a single study each, precluding assessment of consistency. Seventh, the inclusion of dental irrigants (19) and ZOE (20) alongside structural biomaterials introduces conceptual heterogeneity, as the mechanisms governing EB effects in aqueous solutions and cements differ from those in polymeric substrates. Eighth, the search was limited to English-language publications, which may have excluded relevant studies published in German, Japanese, or Portuguese. Ninth, formal assessment of publication bias was not possible. Tenth, five of the 18 included studies (Studies 4, 12, 13, 14, and 17) were identified through hand-searching of reference lists rather than the electronic database search (7, 8, 18, 20, 21); these were published in journals with limited or discontinued indexing in PubMed, Scopus, and Web of Science and overlap in authorship with the review team (see Conflict of Interest). Although they met all inclusion criteria and underwent the same risk-of-bias assessment as the other included studies, their findings should be interpreted with additional caution, and a pre-specified robustness check confirmed that the review's conclusions did not depend on them. Eleventh, the review protocol was registered on the Open Science Framework only after the database search and screening had been completed (retrospective registration); the registry record therefore cannot serve as independent evidence that the review methods were fully specified before the evidence was examined. Twelfth, field experts and study authors were not formally contacted to identify additional or unpublished work, so relevant studies outside the searched databases and reference lists may have been missed.

Finally, the modified GRADE assessment rated all evidence as “Very Low” certainty, which is inherent to the *in vitro* study design and limits the strength of any conclusions drawn.

## 5 Conclusions

This systematic review comprehensively evaluates the effect of electron beam irradiation on dental materials across 18 *in vitro* studies. The following evidence-based conclusions can be drawn.

Regarding the effect of EB irradiation on mechanical properties: EB irradiation at low-to-moderate doses (1–100 kGy) consistently improved flexural strength, fracture toughness, Vickers hardness, and wear resistance of dimethacrylate-based dental polymers and composites across multiple independent studies. These improvements appear to reflect radiation-induced cross-linking within the polymer matrix. ESR evidence of free radical generation, combined with the dose-dependent nature of the response, supports this interpretation.

Regarding materiai responds to electron beam irradiation depends heavily on its composition. BisGMA, UDMA, and TEGDMA-based systems showed consistent benefits, while polyoxymethylene deteriorated and PMMA improved only modestly and only within a narrow dose range. Zirconia is a different case entirely, it underwent crystalline phase transformation, not the polymer-network changes seen in resin-based materials. Given this variation, no single irradiation protocol fits all material types.

Regarding dose-response, optimal dose is not a fixed target-it shifts considerably depending on the material. Nanocomposites responded well at 1–5 kGy, while veneering composites and FRCS required 25–100 kGy to show benefit. Beyond 200 kGy, degradation was the consistent finding across materials. These ranges reflect the specific materials tested in the included studies; extrapolating them to other systems would go beyond what the evidence supports.

Regarding colour stability which is a recurring problem and arguably the most significant obstacle to clinical use. All four studies that measured this outcome reported colour changes exceeding the clinically acceptable threshold (Δ*E* > 3.3). High inorganic filler content (40% SiO_2_) may reduce discolouration, but that finding rests on a single study and needs replication before any conclusions can be drawn.

Regarding bond strength, thiol-phosphate primers used alongside EB irradiation, produced 3–4-fold improvements in alloy-to-resin bond strength. These gains remained stable after thermal cycling-a meaningful result for adhesive prosthodontics, where bond durability under thermal stress is a practical concern.

Regarding biological properties, at optimal doses, EB irradiation can reduce residual monomer leaching and improve the antimicrobial activity of irrigants both potentially useful effects. However, it may also increase the genotoxicity of glass ionomer cements, which is a more serious concern. Given this variation across materials, biocompatibility testing on a material-by-material basis is not optional, it's a prerequisite for any clinical application.

Regarding the certainty of evidence, all conclusions are based on *in vitro* evidence, and under the modified GRADE assessment, that evidence rates as “Very Low” certainty.

Well-designed clinical investigations with standardised irradiation protocols, comprehensive reporting of irradiation parameters (dose, dose rate, number of passes, atmosphere), and long-term follow-up are warranted to determine whether the mechanical property improvements observed *in vitro* translate into meaningful clinical benefits.

## Data Availability

The datasets presented in this study can be found in online repositories. The names of the repository/repositories and accession number(s) can be found in the article/Supplementary Material.

## Supplementary material

The Supplementary Material for this article can be found online at: https://www.frontiersin.org/articles/10.3389/fdmed.2026.1871959/full#supplementary-material

Supplementary Material S1 PRISMA 2020 Checklist.

Supplementary Table S2 Complete electronic search strategy for each database (PubMed, Scopus, Web of Science).

Supplementary Table S3 List of the 29 excluded full-text studies with individual reasons for exclusion.

## References

[1] de Amorim DMG Veríssimo AH Ribeiro AKC De Assunção E Souza RO De Assunção IV Caldas MRGR. Effects of ionizing radiation on surface properties of current restorative dental materials. J Mater Sci: Mater Med. (2021) 32(6):69. 10.1007/s10856-021-06543-5 34117934 PMC8197703

[2] Girard-Perier N Marque SRA Dupuy N Claeys-Bruno M Gaston F Dorey S. Effects of x-rays, electron beam, and gamma irradiation on chemical and physical properties of EVA multilayer films. Front Chem. (2022) 10:888285. 10.3389/fchem.2022.888285 35646817 PMC9131251

[3] Li D Bisel TT Cooley SK Murphy MK Spencer MP Hasan MK. Effects of gamma, electron beam, and x-ray irradiation on the plastic components of a universal bone cement mixer medical device. Radiat Phys Chem. (2024) 223:111971. 10.1016/j.radphyschem.2024.111971

[4] Thongthai P Kitagawa H Iwasaki Y Noree S Kitagawa R Imazato S. Immobilizing bactericides on dental resins via electron beam irradiation. J Dent Res. (2021) 100(10):1055–62. 10.1177/00220345211026569 34301167

[5] Behr M Rosentritt M Faltermeier A Handel G. Electron beam irradiation of dental composites. Dent Mater. (2005) 21(9):804–10. 10.1016/j.dental.2005.01.017 15878195

[6] Behr M Rosentritt M Bettermann K Handel G. Influence of electron beam irradiation on the alloy-to-resin bond strength. Eur J Oral Sci. (2005) 113(5):429–35. 10.1111/j.1600-0722.2005.00236.x 16202032

[7] Hegde MN. Effect of electron beam irradiation on flexural strength of two nanocomposites-an *in vitro* study. Br J Med Med Res. (2014) 4(28):4654–64. 10.9734/bjmmr/2014/10417

[8] Hegde MN Shetty P Bhat R Mathew T. Enhancement of flexural strength of two nano-composites by electron beam irradiation. J Health Allied Sci NU. (2016) 6:14–7. 10.1055/s-0040-1708669

[9] Vaishnavi C Kavitha S Narayanan L. Comparison of the fracture toughness and wear resistance of indirect composites cured by conventional post curing methods and electron beam irradiation. J Conserv Dent. (2010) 13(3):145. 10.4103/0972-0707.71647 21116390 PMC2980611

[10] Kolbeck C Rosentritt M Behr M Schneider S Handel G. Fracture strength and bond capacities of electron irradiated fiber reinforced composites. Dent Mater. (2007) 23(12):1529–34. 10.1016/j.dental.2006.12.008 17395257

[11] Behr M Rosentritt M Dümmler F Handel G. The influence of electron beam irradiation on fibre-reinforced composite specimens. J Oral Rehabil. (2006) 33(6):447–51. 10.1111/j.1365-2842.2005.01583.x 16671992

[12] Faltermeier A Behr M Rosentritt M Handel G. Electron-beam irradiation of experimental denture base polymers. Acta Odontol Scand. (2007) 65(3):171–6. 10.1080/00016350701278781 17514520

[13] Faltermeier A Behr M Reicheneder C Proff P Römer P. Electron-beam irradiation of polymer bracket materials. Am J Orthod Dentofacial Orthop. (2011) 139(1):e7–11. 10.1016/j.ajodo.2009.10.038 21195259

[14] Faltermeier J Simon P Reicheneder C Proff P Faltermeier A. The influence of electron beam irradiation on colour stability and hardness of aesthetic brackets. Eur J Orthod. (2012) 34(4):427–31. 10.1093/ejo/cjr031 21502379

[15] Ito K Nomura A Nomura S Watanabe K. Effects of low-energy electron beam irradiation on flexural properties of self-curing acrylic resin. J Prosthodont Res. (2014) 58(1):55–61. 10.1016/j.jpor.2013.10.004 24393597

[16] Alshamrani A De Souza GM. Effect of ionizing radiation on mechanical properties and translucency of monolithic zirconia. J Biomed Mater Res B Appl Biomater. (2020) 108(3):1068–76. 10.1002/jbm.b.34458 31410974

[17] Tokunaga J Kojima T Kinuta S Wakabayashi K Nakamura T Yatani H. Large-area electron beam irradiation for surface polishing of cast titanium. Dent Mater J. (2009) 28(5):571–7. 10.4012/dmj.28.571 19822988

[18] Hegde MN Shetty SS Shabin S Hegde ND Kumari S Sanjeev G. Genotoxic effects of glass ionomer type-IX cements on human lymphocytes before and after irradiation. Res J Pharm Biol Chem Sci. (2014) 5(2):1056–63.

[19] Shetty V. Antimicrobial activity and stability of electron beam irradiated dental irrigants. J Clin Diagn Res. (2014) 8(11):DC21–4. 10.7860/jcdr/2014/9449.5180 PMC4290238 25584220

[20] Hegde MN Hegde ND Puri A Kumari S Sanjeev G Shetty S. Value addition property of zinc oxide eugenol after electron beam radiation. Res J Pharm Biol Chem Sci. (2013) 4(2):243–6.

[21] Sajnani A Malhotra S Hegde MN. Valuation of leaching of monomers after irradiation to methacrylate-based nano-composites—an *in vitro* study. Res J Pharm Biol Chem Sci. (2014) 5(2):455–63.

[22] Page MJ McKenzie JE Bossuyt PM Boutron I Hoffmann TC Mulrow CD. The PRISMA 2020 statement: an updated guideline for reporting systematic reviews. Br Med J. (2021) 372:n71. 10.1136/bmj.n71 33782057 PMC8005924

[23] Delgado AHS Sauro S Lima AF Loguercio AD Della Bona A Mazzoni A. RoBDEMAT: a risk of bias tool and guideline to support reporting of pre-clinical dental materials research and assessment of systematic reviews. J Dent. (2022) 127:104350. 10.1016/j.jdent.2022.104350 36341980

[24] Sheth VH Shah NP Jain R Bhanushali N Bhatnagar V. Development and validation of a risk-of-bias tool for assessing *in vitro* studies conducted in dentistry: the QUIN. J Prosthet Dent. (2024) 131(6):1038–42. 10.1016/j.prosdent.2022.05.019 35752496

[25] Guyatt GH Oxman AD Vist GE Kunz R Falck-Ytter Y Alonso-Coello P. GRADE: an emerging consensus on rating quality of evidence and strength of recommendations. Br Med J. (2008) 336(7650):924–6. 10.1136/bmj.39489.470347.ad 18436948 PMC2335261

[26] Hooijmans CR De Vries RBM Ritskes-Hoitinga M Rovers MM Leeflang MM IntHout J. Facilitating healthcare decisions by assessing the certainty in the evidence from preclinical animal studies. PLoS One. (2018) 13(1):e0187271. 10.1371/journal.pone.0187271 29324741 PMC5764235

[27] Woźniak L Lompart A Wabiszczewicz M Kosarewicz A Krysiak P. Enhancing the integrity of evidence-based medicine: a literature review on reporting quality in systematic reviews and randomised controlled trials. Qual Sport. (2025) 44:62873. 10.12775/qs.2025.44.62873

[28] Campbell M McKenzie JE Sowden A Katikireddi SV Brennan SE Ellis S. Synthesis without meta-analysis (SWiM) in systematic reviews: reporting guideline. Br Med J. (2020) 368:l6890. 10.1136/bmj.l6890 31948937 PMC7190266

[29] Ravi MS Vijay R Suchetha Kumari N Panchasara C Sanjeev G. Cytotoxic and genotoxic evaluation of three orthodontic bonding adhesives exposed to electron beam radiation. Res J Pharm Biol Chem Sci. (2014) 5(2):1562–71.

[30] Naikwadi AT Sharma BK Bhatt K Mahanwar PA. Gamma radiation processed polymeric materials for high performance applications: a review. Front Chem. (2022) 10:837111. 10.3389/fchem.2022.837111 35360545 PMC8964295

[31] Frison T Seegers WJM Totev D Esteves ACC. Insights on chemical reactions and formation process of electron beam-cured acrylic networks. Macromolecules. (2024) 57(3):1291–301. 10.1021/acs.macromol.3c02085

[32] Mănăilă E Crăciun G Ighigeanu D Lungu IB Dumitru M Stelescu MD. Degradation by electron beam irradiation of some composites based on natural rubber reinforced with mineral and organic fillers. Int J Mol Sci. (2022) 23(13):6925. 10.3390/ijms23136925 35805934 PMC9266345

[33] Bansal N Arora S. Exploring the impact of gamma rays and electron beam irradiation on physico-mechanical properties of polymers and polymer composites: a comprehensive review. Nucl Instrum Methods Phys Res B. (2024) 549:165297. 10.1016/j.nimb.2024.165297

[34] Sharifi S Islam MM Sharifi H Islam R Huq TN Nilsson PH. Electron beam sterilization of poly(methyl methacrylate)—physicochemical and biological aspects. Macromol Biosci. (2021) 21(4):e2000379. 10.1002/mabi.202000379 33624923 PMC8147572

