# Supplementary material for: Dose-dependent effects of electron beam irradiation on dental materials: a systematic review of in vitro evidence
Source: Front Dent Med. 2026 Aug 11;7:1871959. doi: 10.3389/fdmed.2026.1871959 (PMC13503174; doi:10.3389/fdmed.2026.1871959)
Supplement: Supplementary file 2 [file Supplementaryfile2.docx]

**Supplementary Table S2**

| **Database searched** | **Search Query** |
| --- | --- |
| PubMed | (("electron beam irradiation"[Title/Abstract] OR "electron beam radiation"[Title/Abstract] OR "e-beam irradiation"[Title/Abstract] OR "e-beam radiation"[Title/Abstract] OR "electron irradiation"[Title/Abstract] OR "electron beam processing"[Title/Abstract] OR "electron beam treatment"[Title/Abstract] OR "electron beam curing"[Title/Abstract] OR "electron beam sterilization"[Title/Abstract] OR "electron beam sterilisation"[Title/Abstract] OR "accelerated electron"[Title/Abstract] OR "high energy electron"[Title/Abstract] OR "beta irradiation"[Title/Abstract] OR "radiation, ionizing"[MeSH Terms]) AND ("dental material"[Title/Abstract] OR "dental materials"[MeSH Terms] OR "resin composite"[Title/Abstract] OR "composite resin"[Title/Abstract] OR "dental composite"[Title/Abstract] OR "resin-based composite"[Title/Abstract] OR "glass ionomer"[Title/Abstract] OR "glass ionomer cement"[Title/Abstract] OR "PMMA"[Title/Abstract] OR "polymethyl methacrylate"[Title/Abstract] OR "denture base"[Title/Abstract] OR "acrylic resin"[Title/Abstract] OR "dental acrylic"[Title/Abstract] OR "orthodontic bracket"[Title/Abstract] OR "ceramic bracket"[Title/Abstract] OR "fiber reinforced composite"[Title/Abstract] OR "fibre reinforced composite"[Title/Abstract] OR "endodontic sealer"[Title/Abstract] OR "root canal sealer"[Title/Abstract] OR "dental cement"[Title/Abstract] OR "titanium implant"[Title/Abstract] OR "dental implant"[Title/Abstract] OR "zirconia"[Title/Abstract] OR "dental zirconia"[Title/Abstract] OR "dental porcelain"[Title/Abstract] OR "dental ceramic"[Title/Abstract] OR "nanocomposite"[Title/Abstract] OR "dental nanocomposite"[Title/Abstract] OR "sodium hypochlorite"[Title/Abstract] OR "chlorhexidine"[Title/Abstract] OR "irrigant"[Title/Abstract]) AND ("kGy"[Title/Abstract] OR "kilogray"[Title/Abstract] OR "MeV"[Title/Abstract] OR "absorbed dose"[Title/Abstract] OR "radiation dose"[Title/Abstract] OR "dose rate"[Title/Abstract] OR "dose-dependent"[Title/Abstract] OR "irradiation dose"[Title/Abstract])) AND ((english[Filter]) AND (2001:2026[pdat])) |
| Scopus | ( TITLE-ABS-KEY ( "electron beam irradiation" OR "electron beam radiation" OR "e-beam irradiation" OR "e-beam radiation" OR "electron irradiation" OR "electron beam processing" OR "electron beam treatment" OR "electron beam curing" OR "electron beam sterilization" OR "electron beam sterilisation" OR "accelerated electron" OR "high energy electron" OR "beta irradiation" OR "ionizing radiation" OR "ionising radiation" ) ) AND ( TITLE-ABS-KEY ( "dental material" OR "dental materials" OR "resin composite" OR "composite resin" OR "dental composite" OR "resin-based composite" OR "glass ionomer" OR "glass ionomer cement" OR "PMMA" OR "polymethyl methacrylate" OR "denture base" OR "acrylic resin" OR "dental acrylic" OR "orthodontic bracket" OR "ceramic bracket" OR "fiber reinforced composite" OR "fibre reinforced composite" OR "endodontic sealer" OR "root canal sealer" OR "dental cement" OR "titanium implant" OR "dental implant" OR "zirconia" OR "dental zirconia" OR "dental porcelain" OR "dental ceramic" OR "nanocomposite" OR "dental nanocomposite" OR "sodium hypochlorite" OR "chlorhexidine" OR "irrigant" ) ) AND ( TITLE-ABS-KEY ( "kGy" OR "kilogray" OR "MeV" OR "absorbed dose" OR "radiation dose" OR "dose rate" OR "dose-dependent" OR "irradiation dose" ) ) AND PUBYEAR > 2000 AND PUBYEAR < 2027 AND ( LIMIT-TO ( LANGUAGE , "English" ) ) |
| Web of Science | TS=("electron beam irradiation" OR "electron beam radiation" OR "e-beam irradiation" OR "e-beam radiation" OR "electron irradiation" OR "electron beam processing" OR "electron beam treatment" OR "electron beam curing" OR "electron beam sterilization" OR "electron beam sterilisation" OR "accelerated electron" OR "high energy electron" OR "beta irradiation" OR "ionizing radiation" OR "ionising radiation") AND TS=("dental material" OR "dental materials" OR "resin composite" OR "composite resin" OR "dental composite" OR "resin-based composite" OR "glass ionomer" OR "glass ionomer cement" OR "PMMA" OR "polymethyl methacrylate" OR "denture base" OR "acrylic resin" OR "dental acrylic" OR "orthodontic bracket" OR "ceramic bracket" OR "fiber reinforced composite" OR "fibre reinforced composite" OR "endodontic sealer" OR "root canal sealer" OR "dental cement" OR "titanium implant" OR "dental implant" OR "zirconia" OR "dental zirconia" OR "dental porcelain" OR "dental ceramic" OR "nanocomposite" OR "dental nanocomposite" OR "sodium hypochlorite" OR "chlorhexidine" OR "irrigant") AND TS=("kGy" OR "kilogray" OR "MeV" OR "absorbed dose" OR "radiation dose" OR "dose rate" OR "dose-dependent" OR "irradiation dose") and 2026 or 2026 or 2025 or 2024 or 2023 or 2022 or 2021 or 2020 or 2019 or 2018 or 2017 or 2016 or 2015 or 2014 or 2013 or 2012 or 2011 or 2010 or 2009 or 2008 or 2007 or 2006 or 2005 or 2004 or 2003 or 2002 or 2001 (Publication Years) and English (Languages) |
