# Supplementary material for: Dose-dependent effects of electron beam irradiation on dental materials: a systematic review of in vitro evidence
Source: Front Dent Med. 2026 Aug 11;7:1871959. doi: 10.3389/fdmed.2026.1871959 (PMC13503174; doi:10.3389/fdmed.2026.1871959)
Supplement: Supplementary file 3 [file Supplementaryfile3.docx]

**Supplementary Table S3: List of excluded studies with reasons for exclusion**

| **SL. no** | **Authors & Year** | **Study Title** | **Journal** | **Reason for Exclusion** |
| --- | --- | --- | --- | --- |
| 1 | Aljoumaa, K.; Ajji, Z. (2017) | Thermal and mechanical properties of irradiated poly(vinyl chloride)/calcium carbonate composite | Journal of Radioanalytical and Nuclear Chemistry | The material studied was not a dental material |
| 2 | Ananthi et al. (2019) | Conformal electron beam radiotherapy using custom-made step bolus for postmastectomy chest wall irradiation | Cancer Res Ther | The material studied was not a dental material |
| 3 | An et al. (2017) | Preparation and Characterization of Resorbable Bacterial Cellulose Membranes Treated by Electron Beam Irradiation for Guided Bone Regeneration | Int J Mol Sci | The study was conducted in vivo or in animals |
| 4 | Assis et al. (2019) | Ag Nanoparticles/α-Ag(2)WO(4) Composite Formed by Electron Beam and Femtosecond Irradiation as Potent Antifungal and Antitumor Agents | Sci Rep | Electron beam irradiation was not used to modify a dental material |
| 5 | Attin et al. (2001) | Antibacterial properties of electron beam-sterilized Gutta-Percha cones | J Endod | Electron beam irradiation was not used to modify a dental material |
| 6 | BahreyniToossi et al. (2016) | Evaluation of the effect of tooth and dental restoration material on electron dose distribution and production of photon contamination in electron beam radiotherapy | Australas Phys Eng Sci Med | The study did not assess post-irradiation properties of the dental material |
| 7 | Brès et al. | HREM study of irradiation damage in human dental enamel crystals | Ultramicroscopy | The material studied was not a dental material |
| 8 | Cairns et al. (2012) | The potential of electron beam radiation for simultaneous surface modification and bioresorption control of PLLA | Journal of Biomedical Materials Research Part A | The material studied was not a dental material |
| 9 | Choi et al. (2013) | Characterization and antimicrobial property of poly(acrylic acid) nanogel containing silver particle prepared by electron beam | Int J Mol Sci | Electron beam irradiation was not used to modify a dental material |
| 10 | Dånmark et al. (2011) | In vitro and in vivo degradation profile of aliphatic polyesters subjected to electron beam sterilization | Acta Biomater | Electron beam irradiation was not used to modify a dental material |
| 11 | Dogan et al. (2013) | The effects of therapeutic x-ray doses on mechanical, chemical and physical properties of poly methyl methacrylate | Acta Odontologica Scandinavica | The intervention was not electron beam irradiation |
| 12 | Ergun et al. (2020) | A comparative study of heat-cured and gamma-cured fiber-reinforced denture-base acrylic resins: Residual monomer and flexural strength | Polymers & Polymer Composites | The intervention was not electron beam irradiation |
| 13 | Faga et al. (2021) | Electron-Beam-Induced Grafting of Chitosan onto HDPE/ATZ Composites for Biomedical Applications | Polymers (Basel) | The material studied was not a dental material |
| 14 | Fattibene et al. (2004) | Dosimetric response of tooth enamel to 14 Mev neutrons | Radiat Environ Biophys | The intervention was not electron beam irradiation |
| 15 | Hashemizadeh et al. (2024) | Calculation of Dose Perturbation in Radiotherapy of Head and Neck Tumors Due to the Presence of Dental Implants: A Monte Carlo Study | Frontiers in Biomedical Technologies | The intervention was not electron beam irradiation |
| 16 | Hegde et al. (2019) | Protection of wear resistance behaviour of enamel against electron beam irradiation | BDJ Open | The material studied was not a dental material |
| 17 | Hussain et al. (2024) | Investigation in vitro the effect of X-rays, gamma rays and beta particles on the physical and structural characteristics of human teeth | AIP Conference Proceedings | The intervention was not electron beam irradiation |
| 18 | Ichimiya et al. (2013) | Growth of high-quality CuCl thin films by a technique involving electron-beam irradiation | Journal of Crystal Growth | The material studied was not a dental material |
| 19 | Kim et al. (2015) | Electron beam effect on biomaterials I: focusing on bone graft materials | Biomater Res | The study was conducted in vivo or in animals |
| 20 | Labus et al. (2022) | Influence of electron beam irradiation on extracellular matrix of the human allogeneic skin grafts | Journal of Biomedical Materials Research Part B | The material studied was not a dental material |
| 21 | Lee et al. (2017) | The Efficacy of Electron Beam Irradiated Bacterial Cellulose Membranes as Compared with Collagen Membranes on Guided Bone Regeneration in Peri-Implant Bone Defects | Materials (Basel) | The study was conducted in vivo or in animals |
| 22 | Lee et al. (2024) | Fabrication and Characterization of Electrospun Cu-Doped TiO(2) Nanofibers and Enhancement of Photocatalytic Performance | Polymers (Basel) | The material studied was not a dental material |
| 23 | Lian et al. (2009) | Ion beam-induced amorphous-to-tetragonal phase transformation and grain growth of nanocrystalline zirconia | Nanotechnology | The intervention was not electron beam irradiation |
| 24 | Mirhashmi et al. (2020) | Optimized Er: YAG Laser Irradiation Distance to Achieve the Strongest Bond Strength Between Orthodontic Brackets and Zirconia-Ceramics | Journal of Lasers in Medical Sciences | The intervention was not electron beam irradiation |
| 25 | Ninaya et al. (2016) | Development and Evaluation of Surface Modified Poly (lactic acid) Microsphere via Irradiation Techniques for Drug Delivery System | 5th International Conference on Recent Advances in Materials | The material studied was not a dental material |
| 26 | Nishi et al. (2011) | Effects of Electron Beam Irradiation on Peeling Resistance of Laminated Sheet of High Strength Polypropylene (PP) and Bio-Adaptable Polydimethylsiloxane (PDMS) | Materials Transactions | The material studied was not a dental material |
| 27 | Nishi et al. (2012) | Effects of Electron Beam Irradiation on Adhesive Force of Laminated Sheet of High Strength Polytetrafluoroethylene (PTFE) and Bio-Adaptable Polydimethylsiloxane (PDMS) | Materials Transactions | The material studied was not a dental material |
| 28 | Sarı et al. (2018) | Efficacy of Various Pretreatments on the Bond Strength of Denture Teeth to Denture Base Resins | Photomedicine and Laser Surgery | The intervention was not electron beam irradiation |
| 29 | Tiznado-Orozco et al. (2015) | Wettability modification of human tooth surface by water and UV and electron-beam radiation | Mater Sci Eng C Mater Biol Appl | The material studied was not a dental material |
